# Metabolomic profiling combined with network analysis of serum pharmacochemistry to reveal the therapeutic mechanism of Ardisiae Japonicae Herba against acute lung injury

**DOI:** 10.3389/fphar.2023.1131479

**Published:** 2023-07-24

**Authors:** Xiao-Xiao Han, Yan-Ge Tian, Wen-Jing Liu, Di Zhao, Xue-Fang Liu, Yan-Ping Hu, Su-Xiang Feng, Jian-Sheng Li

**Affiliations:** ^1^ College of Pharmacy, Henan University of Chinese Medicine, Zhengzhou, Henan, China; ^2^ Collaborative Innovation Center for Chinese Medicine and Respiratory Diseases Co-constructed by Henan Province and Education Ministry of P. R. China, Zhengzhou, Henan, China; ^3^ Academy of Chinese Medical Sciences, Henan University of Chinese Medicine, Zhengzhou, Henan, China; ^4^ The First Affiliated Hospital, Henan University of Chinese Medicine, Zhengzhou, Henan, China

**Keywords:** Ardisiae Japonicae Herba, serum pharmacochemistry, network analysis, metabolomics, acute lung injury

## Abstract

**Introduction:** Acute lung injury (ALI) is a common and devastating respiratory disease associated with uncontrolled inflammatory response and transepithelial neutrophil migration. In recent years, a growing number of studies have found that Ardisiae Japonicae Herba (AJH) has a favorable anti-inflammatory effect. However, its serum material basis and molecular mechanism are still unknown in ALI treatment. In this study, metabolomics and network analysis of serum pharmacochemistry were used to explore the therapeutic effect and molecular mechanism of AJH against lipopolysaccharide (LPS)-induced ALI.

**Methods:** A total of 12 rats for serum pharmacochemistry analysis were randomly divided into the LPS group and LPS + AJH-treated group (treated with AJH extract 20 g/kg/d), which were administered LPS (2 mg/kg) by intratracheal instillation and then continuously administered for 7 days. Moreover, 36 rats for metabolomic research were divided into control, LPS, LPS + AJH-treated (5, 10, and 20 g/kg/d), and LPS + dexamethasone (Dex) (2.3 × 10^−4^ g/kg/d) groups. After 1 h of the seventh administration, the LPS, LPS + AJH-treated, and LPS + Dex groups were administered LPS by intratracheal instillation to induce ALI. The serum pharmacochemistry profiling was performed by UPLC-Orbitrap Fusion MS to identify serum components, which further explore the molecular mechanism of AJH against ALI by network analysis. Meanwhile, metabolomics was used to select the potential biomarkers and related metabolic pathways and to analyze the therapeutic mechanism of AJH against ALI.

**Results:** The results showed that 71 serum components and 18 related metabolites were identified in ALI rat serum. We found that 81 overlapping targets were frequently involved in AGE-RAGE, PI3K-AKT, and JAK-STAT signaling pathways in network analysis. The LPS + AJH-treated groups exerted protective effects against ALI by reducing the infiltration of inflammatory cells and achieved anti-inflammatory efficacy by significantly regulating the interleukin (IL)-6 and IL-10 levels. Metabolomics analysis shows that the therapeutic effect of AJH on ALI involves 43 potential biomarkers and 14 metabolic pathways, especially phenylalanine, tyrosine, and tryptophan biosynthesis and linoleic acid metabolism pathways, to be influenced, which implied the potential mechanism of AJH in ALI treatment.

**Discussion:** Our study initially elucidated the material basis and effective mechanism of AJH against ALI, which provided a solid basis for AJH application.

## Graphical Abstract

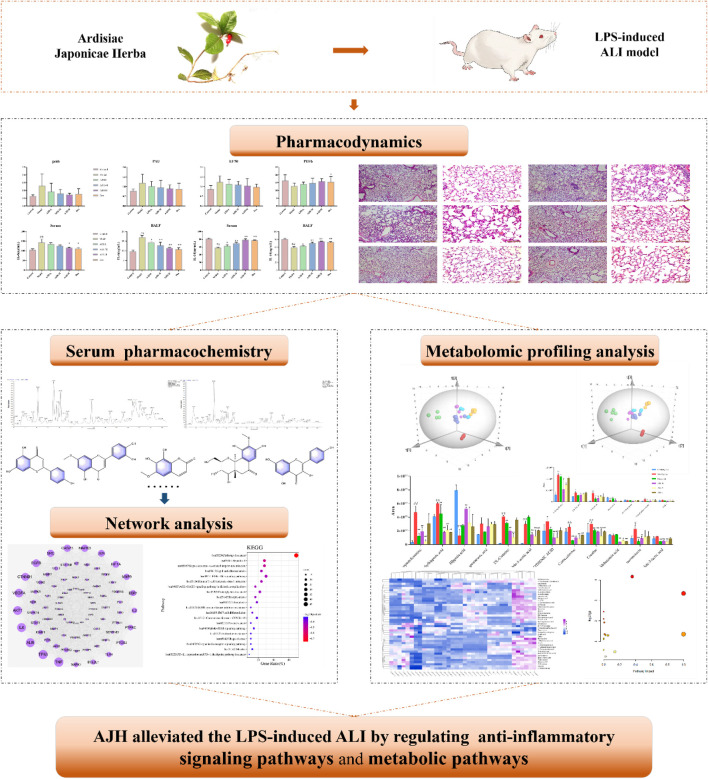

## 1 Introduction

Acute lung injury (ALI), a common and devastating respiratory disease, is induced by inhalation injury, acute pneumonia, trauma, sepsis, pulmonary edema, and acute pancreatitis (Savino et al., 2019; Bin et al., 2022; Ying et al., 2022). ALI is the leading cause of morbidity and mortality in intensive care units (SeungHye and Mallampalli, 2015; Huang et al., 2018), and the mortality rate is 35%–55% (Levitt et al., 2013). The pathological characteristics are associated with excessive immune cell activation, oxidative stress, inflammation, hypoxemia, bilateral lung infiltration, and electrolyte disturbances (Jiang et al., 2020; liu et al., 2021; Ying et al., 2022). ALI is considered an acute lung disease and has severe inflammatory response outbreaks in the lung accompanied by widespread damage to epithelial and endothelial cells, macrophage activation, and neutrophil infiltration (Wu et al., 2022; Zhang et al., 2022). Zhang et al. (2019) demonstrated that dexmedetomidine can inhibit the inflammatory response by regulating GSK-3β/STAT3-NF-κB. In addition, several studies demonstrated that some Chinese medicines (CMs) improve ALI by regulating relevant inflammatory cytokines and pathways. Scutellariae Radix can improve ALI by reducing the expression of nitric oxide (NO), tumor necrosis factor alpha (TNF-α), interleukin (IL)-6, and IL-8 (Hu et al., 2020). In addition, *Sarcandra glabra* combined with lycopene ameliorates histopathological injuries and decreases the levels of TNF-α and IL-6. Mitogen-activated protein kinase (MAPK) and transcription factor NF-kB were activated in lipopolysaccharide (LPS)-induced ALI rats, which exhibit a significant effect in protecting and improving LPS-induced ALI rats (Liu and Chen, 2016). Therefore, deregulating inflammatory response may play a significant role and be available for ALI treatment.

Ardisiae Japonicae Herba (AJH, *Ardisia japonica* (Thunb.) Blume), having a slightly bitter taste, belongs to the genus Ardisia and family Primulaceae. AJH converges the lung and liver meridians and has detoxification effects and activates blood circulation, which is recorded in the Materia Medica based on Chinese medical theory. On the basis of its anti-inflammatory (Liu et al., 2009), anti-cancer (Chang et al., 2007; Li et al., 2012), and anti-viral effects (Tien et al., 2007), AJH is widely used to improve chronic bronchitis (Xi, 2006) and hepatoma carcinoma (Gong et al., 2021). Cao et al. (2021) demonstrated that flavonoids in AJH showed an anti-inflammatory effect by regulating the levels of TNF-α and IL-1β, which could inhibit the proliferation and activation of hepatic stellate cells, and affect the immune function. In our previous study, we found that three components in AJH, namely, bergenin, luteolin, and kaempferol, decreased the IL-6 and matrix metalloproteinase (MMP) 9 levels, which showed a significant anti-inflammatory effect in a TNF-α-induced A549 cell model (Feng et al., 2022). Therefore, it can be deduced that AJH was effective against ALI through the anti-inflammatory mechanism. In order to confirm this hypothesis, we investigated the therapeutic effect and molecular mechanism of AJH on LPS-induced ALI rats by network analysis and metabolomics.

Due to the complexity of serum components of CMs, network analysis is often applied to study the pharmacological action and molecular mechanism of components or serum components by computer algorithms and network databases, including Metascape, GeneCards, and STRING (Societies, 2021; Han et al., 2022; Xiong et al., 2022). Moreover, metabolomics can comprehensively identify and quantify endogenous metabolites to systematically investigate the metabolic response and pathway by multivariate statistical analysis, which has been widely used in disease diagnosis and toxicology (Liu and Chen, 2016). Therefore, we integrate network analysis and metabolomics to investigate the material basis and the anti-inflammatory molecular mechanism of AJH against ALI. First, the serum components of AJH in the ALI model rat were identified by UPLC-Orbitrap Fusion MS. Subsequently, we further systematically hypothesized and incorporated the potential therapeutic targets and molecular mechanism based on the network analysis. Finally, the therapeutic mechanisms of AJH against ALI were evaluated by biochemical indexes and histopathology of the lung. Furthermore, the serum metabolic profile was confirmed to analyze the metabolic pathways.

## 2 Materials and methods

### 2.1 Materials

AJH (*Ardisia japonica* (Thunb.) Blume) was purchased from Zhangshu Tianqitang Chinese medicine Co., Ltd (Jiangxi, China; batch: 201909001) and authenticated by Dr. Suiqing Chen, Henan University of Chinese Medicine. The voucher specimen was stored at the Scientific Research Center, Henan University of Chinese Medicine, Zhengzhou. The reference standards of methyl salicylate (batch: CHB190925), kaempferol (batch: CHB190127), embelin (batch: CHB190829), hydroxygenkwanin (batch: CHB180611), rapanone (batch: CHB190723), (−)-epigallocatechin gallate (batch: CHB180307), myricitrin (batch: CHB180611), 3,4-dimethoxybenzoic acid (batch: CHB190820), ethyl gallate (batch: CHB180116), myricetin (batch: CHB180614), astilbin (batch: CHB190107), apigenin (batch: CHB180103), and (−)-epicatechin gallate (batch: CHB180305) (purity ≥98%) were obtained from Chengdu Chroma-Biotechnology Co., Ltd (Chengdu, China). Quercetin (batch: MUST-20101104, purity ≥99.35%) was purchased from Chengdu Must Bio-technology Co., Ltd. Bergenin (batch: 111532–201604, purity ≥94.1%) was obtained from National Institutes for Food and Drug Control (Beijing, China). HPLC-grade methanol and formic acid of mass grade were purchased from Thermo Fisher (United States). Ultra-pure water was prepared using a Milli-Q purification system (Millipore, Merck).

Dexamethasone acetate tablets were purchased from Anhui Jintaiyang Pharmaceutical Company Ltd (batch: 2104082; specification: 0.75 mg * 100 pills; usage and dosage: 0.75–3 mg/times and 2–4 times/day; experimental usage and dosage: 0.75 mg/times and 3 times/day). Whole-value grain feedstuff was purchased from SPF (Beijing) Biotechnology Company Ltd (SCXK (Jing) 2019–0010). LPS (batch: 039M4004V) was obtained from Sigma-Aldrich Company Ltd. The levels of IL-6 and IL-10 were quantified by the enzyme-linked immunosorbent assay (ELISA) kits obtained from Wuhan Boster Biological Technology Company Ltd.

### 2.2 Preparation of sample solution

#### 2.2.1 AJH sample preparation

An accurately weighed sample (500 g) of AJH was extracted twice with 5,000 mL ethanol–water (7:3, v/v) under reflux for 1 h each time. The AJH extract was concentrated under reduced pressure to 2.4 g/mL.

#### 2.2.2 Standard solution preparation

A total of 15 reference standards, such as methyl salicylate, kaempferol, and embelin, were accurately weighed, dissolved in methanol to prepare a mixed stock solution with appropriate concentration, and stored at 4°C until further use.

### 2.3 Animals and treatments

**FIGURE 1:**
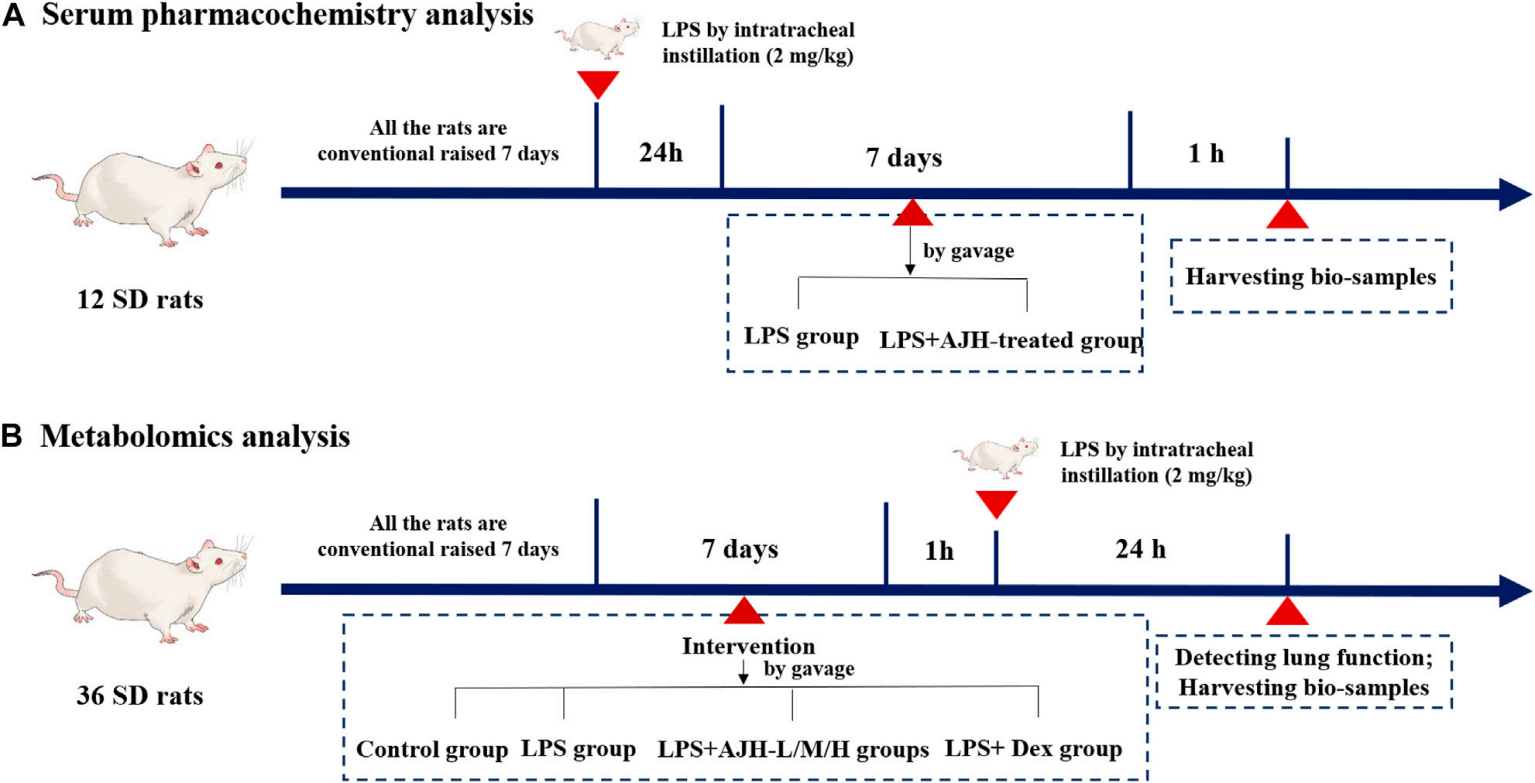
Process of animal grouping and treatment in serum pharmacochemistry and metabolomics tests. (**(A)** serum pharmacochemistry analysis; **(B)** metabolomics analysis).

Male Sprague–Dawley rats (body weight 200 ± 20 g) (NO.: 1107261911004350) were purchased from the Animal Experimental Center of Huaxing (Henan, China, SCXK(Yu) 2019–0002). The experimental protocol was reviewed and approved by the Experimental Animal Care and Ethics Committee of Henan University of Chinese Medicine (Henan, China, SYXK(Yu) 2020–0004). The rats were housed at the Center of Experimental Animals in Henan University of Chinese Medicine, maintained under a 12 h light–dark cycle, temperature of 23°C ± 2°C, and humidity of 50%–60%, with free access to water and food (Han et al., 2022). For serum pharmacochemistry analysis, 12 rats were randomly divided into two groups (*n* = 6 each): LPS (treated with 10 mL/kg/d distilled water by gavage) and LPS + AJH-treated groups (treated with AJH extract 20 g/kg/d by gavage). The rats in the groups were administered LPS by intratracheal instillation (2 mg/kg) to induce ALI. After 24 h of LPS exposure, the LPS and LPS + AJH-treated groups were continuously administered for 7 days (Figure 1A). After 1 h of the last administration, the serum samples were collected, separated by centrifugation at 3,000 rpm for 15 min, and then stored at −80°C. A total of 36 rats for metabolomics analysis were divided into control (treated with 10 mL/kg/d distilled water by gavage), LPS (treated with 10 mL/kg/d distilled water by gavage), LPS + AJH-low (LPS + AJH-L) (treated with AJH extract 5 g/kg/d by gavage), LPS + AJH-medium (LPS + AJH-M) (treated with AJH extract 10 g/kg/d by gavage), LPS + AJH-high (LPS + AJH-H) (treated with AJH extract 20 g/kg/d by gavage), and LPS + dexamethasone (LPS + Dex) (treated with Dex 2.3 × 10^−4^ g/kg/d by gavage). The LPS + AJH-treated and LPS + Dex groups were continuously administered for 7 days. After 1 h of the last administration, the LPS, LPS + AJH-treated, and LPS + Dex groups were anesthetized by intraperitoneal injection of 20% urethane and administered LPS by intratracheal instillation (2 mg/kg) to establish an ALI model (Figure 1B). Furthermore, the LPS doses were selected as previously described (Hu et al., 2020). Subsequently, 24 h after modeling, 36 rats for metabolomic research were tested for lung function and then euthanized after anesthesia to harvest bio-samples including serum, lung tissues, and bronchoalveolar lavage fluid (BALF). Furthermore, BALF was centrifuged for 15 min at 3,000 rpm and stored at −80°C for evaluation of the inflammatory cytokine levels. The serum samples of metabolomics were collected and separated by centrifugation at 3,000 rpm for 15 min and then stored at −80°C and were used to evaluate the inflammatory cytokine levels and metabolomics analysis.

### 2.4 Pathological parameters

According to related studies, several pathological parameters were investigated in our study, including histopathologic evaluation stained with hematoxylin and eosin (H&E), lung wet/dry (W/D) ratio, indexes of the thymus and spleen, lung function, and the levels of inflammatory cytokines (IL-6 and IL-10) in serum and BALF, to assess AJH intervention effects for the ALI model.

### 2.5 Serum sample preparation

For serum pharmacochemistry analysis, 1,500 µL methanol solution was mixed with 500 µL serum sample, vortexed for 6 min, and then centrifuged at 14,000 rpm for 15 min at 4°C. The supernatant was collected and dehydrated under vacuum conditions. Furthermore, dried samples were mixed with 200 μL methanol–water (1:1, v/v), followed by 6 min vortex and 15 min centrifugation (14,000 rpm, 4°C). The supernatant was then analyzed by UPLC-Orbitrap Fusion MS.

In addition, 200 µL rat serum was added to 600 µL methanol, vortexed for 6 min, centrifuged for 15 min at 14,000 rpm and 4°C, and evaporated to dryness. Next, 100 μL methanol–water (1:1, v/v) was added after a vortex and centrifugation according to similar conditions, and the supernatant was analyzed by UPLC-Orbitrap Fusion MS for metabolomics analysis.

### 2.6 UPLC-Orbitrap Fusion MS conditions of serum pharmacochemistry and metabolomics analysis

For serum pharmacochemistry analysis, the sample was separated for analysis on the UPLC-Orbitrap Fusion MS (Thermo Scientific, United States) equipped with a UPLC column (Hypersil GOLD 100 mm × 2.1 mm, 3 μm). The mobile phase system was composed of methanol (A) and 0.1% formic acid in water (B). The flow rate was controlled at 0.2 mL/min with a gradient program of 0–2 min, 93–70%B; 2–12 min, 70–30%B; 12–16 min, 30–20%B; and 16–27 min, 20–0%B. The column temperature and the injection volume were preset at 30°C and 5 μL, respectively. Mass spectral data acquisition was performed on Orbitrap Fusion MS equipped with an electrospray ionization source (ESI) in positive and negative ion modes. The optimal conditions of the ion source was set as follows: evaporation temperature, 275°C; sheath gas, 35 Arb; spray voltage, 3.50 Kv (in the positive ion mode) and −2.50 Kv (in the negative ion mode); auxiliary gas, 7 Arb; capillary temperature, 300°C. Scan mode: full mass (±) (resolution: 120,000); scan range: m/z 120–1,200.

For metabolomics analysis, the mobile phase system was composed of methanol (A) and 0.1% formic acid in water (B). The flow rate was controlled at 0.2 mL/min with a gradient program of 0–5 min, 93–70%B; 5–13 min, 70–48%B; 13–14 min, 48–6%B; 14–17 min, 6–3%B; and 17–20 min, 3–0%B. The temperature of the column was maintained at 30°C, and the injection volume was 5 μL. The scan range of Orbitrap Fusion MS was preset at m/z 100–1,000. The other parameters of UPLC and Orbitrap Fusion MS were consistent with serum pharmacochemistry analysis conditions.

### 2.7 Network analysis based on serum pharmacochemistry in ALI rats

The raw data of serum pharmacochemistry analysis were preprocessed using Compound Discoverer 3.3 software to identify the structure of serum components and further determine the aforementioned components by comparing the fragmentation pathways in Thermo Scientific™ Mass Frontier 7.0 software. The targets of the serum components were predicted from the Swiss Target Prediction database (http://www.swisstargetprediction.ch/). The GeneCards database (https://www.genecards.org/) and the Online Mendelian Inheritance in Man (OMIM) database (https://omim.org/) were used to collect the ALI-associated targets, which were searched using the keywords “acute lung injury”. To find 81 overlapping targets of AJH treatment for ALI from Venny obtained using the bioinformatics analysis platform (http://www.bioinformatics.com.cn/), which were entered into the STRING database (https://cn.string-db.org/cgi/input.pl) for protein–protein interaction (PPI) analysis, a TSV file was downloaded. The AJH–serum component–target–ALI network and PPI network were constructed using Cytoscape 3.2.1 software (Cytoscape Consortium, National Institute of General Medical Sciences, United States). Then, enrichment analysis was carried out in the Metascape database (https://metascape.org/) to predict and analyze Gene Ontology (GO) and the related signaling pathways.

### 2.8 Metabolomics data processing based on multivariate data analysis

Principal component analysis (PCA) was used to visualize the global chemical variations among the control, LPS, LPS + AJH-L, LPS + AJH-M, LPS + AJH-H, and LPS + Dex groups, while partial least squares-discriminant analysis (PLS-DA) was utilized to discover the potential biomarkers, which were defined as the components displaying a variable importance in projection (VIP) > 1.0 in the current work. Then, the potential biomarkers were identified by comparing the Human Metabolome Database (http://www.hmdb.ca/) and the Kyoto Encyclopedia of Genes and Genomes (http://www.kegg.ca/). The potential biomarkers were inputted into MetaboAnalyst 5.0 to reveal the related metabolic pathway. The metabolic pathways with an impact value >0.10 were considered to be the potential target pathway.

### 2.9 Statistical analysis

All data were expressed as the mean ± standard deviation, and statistical analysis was carried out by one-way analysis of variance using SPSS Statistics 26.0. Least significant difference analysis was applied to groups that conformed to the homogeneity test of variance, while Dunnett’s T3 test was performed for groups inconsistent with the homogeneity test of variance. α < 0.05 was considered statistically significant. All metabolomics data preprocessed using Compound Discoverer 3.3 and Mass Frontier 7.0. were inputted into SIMCA 14.1 software for the multivariate statistical analysis, including PCA and PLS-DA.

## 3 Results

### 3.1 Biochemical parameters and histopathologic analysis

**FIGURE 2:**
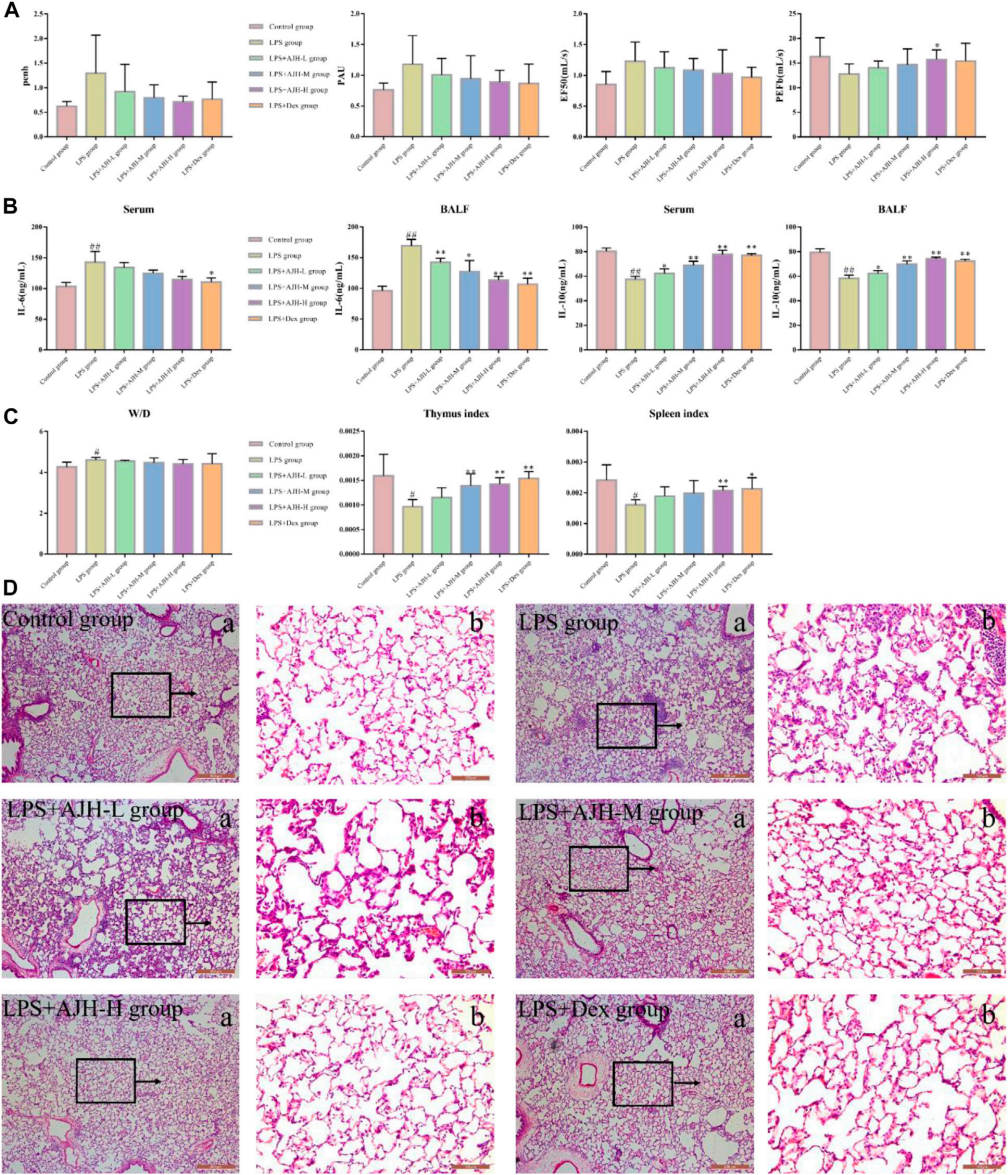
Effect of AJH intervention on ALI based on general characteristics, biochemical analysis, and pathological changes. **(A)** Measurement of the relevant indexes of lung function analysis in rats, including penh, PAU, EF50, and PEFb. **(B)** AJH regulated the expression levels of IL-6 and IL-10 in serum and BALF. **(C)** Lung W/D ratio and thymus and spleen indexes. ^#^ *p* < 0.05, ^##^ *p* < 0.01 vs the control group; *p < 0.05, **p < 0.01 vs the LPS group. **(D)** Histopathological changes in lung tissue and effects of AJH on the ALI model rat ((a) magnification ×50, (b) magnification ×200).

Compared with the control group, IL-6 in serum and BALF was significantly upregulated and IL-10 was downregulated in the LPS group, indicating successful modeling of ALI (*p* < 0.01), as shown in Supplementary Table S1. The level of IL-6 in serum and BALF was decreased; however, the IL-10 expression significantly increased in LPS + AJH groups and LPS + Dex group compared with the LPS group (Figure 2B). Results of IL-6 and IL-10 levels indicated that the protective effects of AJH showed obvious improvement. In addition, AJH groups improved the parameters of lung function by regulating bronchoconstriction and airway resistance, with no significant difference, as shown in Figure 2A and Supplementary Table S2. In order to further identify the therapeutic effect of AJH, the lung W/D ratio and indexes of the thymus and spleen were detected and calculated (Figure 2C and Supplementary Table S3). Compared with the control group, the indexes of the thymus and spleen were decreased in the LPS group (*p* < 0.05 and 0.01). The thymus and spleen indexes of LPS + AJH-H and LPS + Dex groups were significantly increased compared with those of the LPS group (*p* < 0.05 and 0.01). In addition, compared with the control group, the W/D ratio in the LPS group was markedly increased, indicating severe edema (*p* < 0.01). The LPS + AJH-treated groups could improve lung, thymus, and spleen injury. Pathological results further indicated that the LPS group showed distinct histological changes, including alveolar ectasia, alveolar fusion, airway wall thickening, and infiltration of a mass of inflammatory cells into alveolar spaces. Pathological changes were alleviated in the LPS + AJH-treated groups (Figure 2D) compared with the LPS group. All these results showed that the ALI model was successfully established, and the LPS + AJH-L, LPS + AJH-M, and LPS + AJH-H groups have a therapeutic effect on ALI (lung function, lung W/D ratio, indexes of the thymus and spleen, and histological changes), which appeared to be dose-dependent. The AJH-treated groups could also reduce the inflammation on ALI. The anti-inflammatory activity of LPS + AJH-H and LPS + AJH-M groups was better than that of the LPS + AJH-L group.

### 3.2 Characterization and identification of serum components in the ALI rat model

**FIGURE 3:**
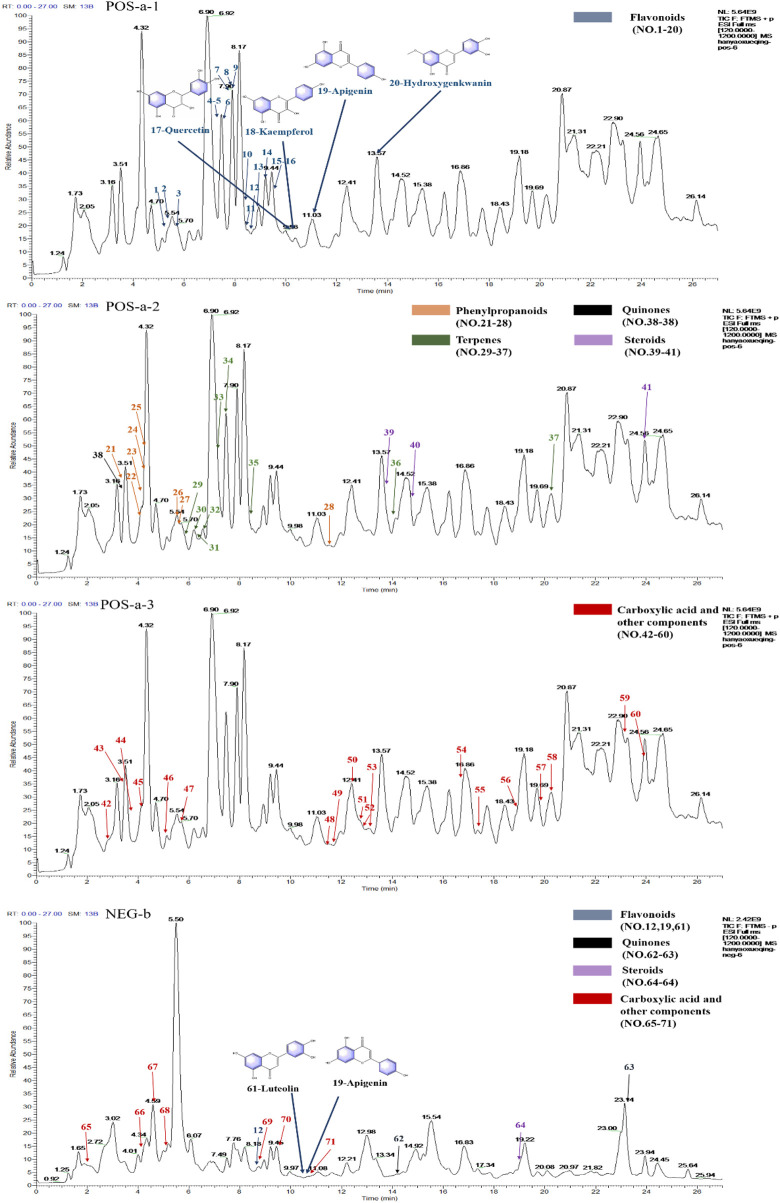
Total ion chromatograms (TICs) of serum pharmacochemistry of AJH in ALI model rats; flavonoids (21 in total) are marked in blue, phenylpropanoids (eight in total) are marked in orange, terpenes (nine in total) are marked in green, quinones (three in total) are marked in black, steroids (4 in total) are marked in purple, and carboxylic acid and other components (26 in total) are marked in red. (a1–3. positive ion mode; b. negative ion mode).

The UPLC-Orbitrap Fusion MS, an instrument with high sensitivity and accuracy, was employed to identify the serum components in the ALI rat model. The total ion chromatogram (TIC) is shown in Figure 3. A total of 71 serum components and 18 related metabolites were identified in ALI rats, and the details including name, retention time, and fragments ions are presented in Tables 1, 2. Among them, 21 components, including kaempferol, myricitrin, and hydroxygenkwanin, were classified as flavonoids; eight components, including bergenin, fraxetin, and scopoletin, were identified as phenylpropanoids; and nine terpenes and three quinones, four steroids, 26 carboxylic acids, and other components were also observed. In addition, 15 serum components were identified by comparing with the reference standards.

**TABLE 1:** Details about 71 serum components of AJH in ALI rats.

No.	Identification	Formula	Experimental MS (m/z)	Measured MS (m/z)	Ion mode	Rt (min)	NL	UPLC-Orbitrap Fusion MS/MS fragments (m/z)
1	Cianidanol	C_15_H_14_O_6_	291.08631	291.08603	[M + H]^+^	5.22	8.21E+06	139.03833,123.04332,119.04829
2	Epigallocatechin gallate	C_22_H_18_O_11_	459.09219	459.09281	[M + H]^+^	5.47	1.29E+05	229.04925,163.03815,153.01743,139.03825,135.04340,125.02317
3	Epigallocatechin	C_15_H_14_O_7_	307.08123	307.08092	[M + H]^+^	5.74	5.27E+05	229.04886,169.04962,163.03816,139.03824,127.03838,111.04329
4	Myricitrin	C_21_H_20_O_12_	465.10275	465.10231	[M + H]^+^	7.53	3.71E+05	449.10737,331.04511,319.04493,127.03986
5	Hyperoside	C_21_H_20_O_12_	465.10275	465.10231	[M + H]^+^	7.53	3.71E+05	361.05469,318.03699,165.01767,153.01761
6	Taxifolin	C_15_H_12_O_7_	305.06558	305.06523	[M + H]^+^	7.57	2.40E+06	167.03305,161.02266,153.01746,151.03816,149.02257,123.04352
7	Cynaroside	C_21_H_20_O_11_	449.10784	449.10804	[M + H]^+^	7.8	1.35E+06	341.06534,287.05411,285.03873,153.01759,137.02287
8	Rutin	C_27_H_30_O_16_	611.16066	611.16063	[M + H]^+^	7.85	3.73E+05	434.31986,303.04828,153.01752,151.03825
9	Astilbin	C_21_H_22_O_11_	451.12349	451.12301	[M + H]^+^	8	2.10E+05	305.06528,287.05326,247.05983,183.02783,177.01764,165.01741
10	Apigenin-7-O-rutinoside	C_27_H_30_O_14_	579.17083	579.17279	[M + H]^+^	8.33	4.95E+06	271.06042,251.09155,167.07050
11	Epicatechin gallate	C_22_H_18_O_10_	443.09727	443.09705	[M + H]^+^	8.47	1.15E+06	319.04459,163.03907,149.02328,137.05978,135.04411,127.03901
12	Myricetin	C_15_H_10_O_8_	319.04484	319.04459	[M + H]^+^	8.55	1.59E+06	194.02125,193.01260,165.01741,153.01741,139.03819,137.02272
	Myricetin	C_15_H_10_O_8_	317.03029	317.03061	[M-H]^-^	8.58	1.20E+06	289.03586,237.11314,219.10246,178.99829,151.00334,137.02409
13	Luteolin-7-o-rutinoside	C_27_H_30_O_15_	595.16575	595.16514	[M + H]^+^	8.79	1.09E+05	395.07574,287.05328,163.05959,145.04890,135.04378
14	Eriodictyol	C_15_H_12_O_6_	289.07066	289.07045	[M + H]^+^	9.17	6.38E+05	163.03786,153.01738,137.05890,123.04350
15	Apigenin-7-O-glucoside	C_21_H_20_O_10_	433.11292	433.113	[M + H]^+^	9.86	1.24E+05	287.05341,165.01737,153.01752,137.02246
16	Afzelin	C_21_H_20_O_10_	433.11292	433.113	[M + H]^+^	9.86	1.24E+05	287.05341,165.01737,153.01752,137.02246
17	Quercetin	C_15_H_10_O_7_	303.04993	303.04997	[M + H]^+^	10.18	3.50E+06	195.02780,165.01746,153.01744,149.02246,137.02271
18	Kaempferol	C_15_H_10_O_6_	287.05501	287.05489	[M + H]^+^	10.72	3.41E+06	287.05493,269.04440,259.05978,231.06528,153.01827
19	Apigenin	C_15_H_10_O_5_	271.0601	271.05987	[M + H]^+^	11.84	3.03E+06	163.03876,153.01738,145.02841,121.02782,119.04863
	Apigenin	C_15_H_10_O_5_	269.04555	269.04553	[M-H]^-^	11.86	1.13E+06	269.04486,253.04996,117.03370
20	Hydroxygenkwanin	C_16_H_12_O_6_	301.07066	301.07037	[M + H]^+^	13.37	1.23E+07	283.05939,167.03323,107.04848
21	Bergenin	C_14_H_16_O_9_	329.08671	329.08684	[M + H]^+^	3.31	1.03E+07	293.06567,275.05524,263.05508,251.05508,237.03936
22	Fraxetin	C_10_H_8_O_5_	209.04445	209.04461	[M + H]^+^	4.07	2.35E+05	194.01999,181.04860,166.02537,163.03799,153.05370,149.02237
23	4-Methoxycinnamic acid	C_10_H_10_O_3_	179.07027	179.0704	[M + H]^+^	4.12	2.93E+06	161.05899,133.06421,118.04059,109.06401,105.06937
24	Methyl cinnamate	C_10_H_10_O_2_	163.07536	163.07558	[M + H]^+^	4.18	5.75E+06	145.06491,131.04929,107.04924
25	(2E)-3-(2,3-Dihydro-1,4-benzodioxin-6-yl)acrylic acid	C_11_H_10_O_4_	207.06518	207.06514	[M + H]^+^	4.22	2.33E+05	163.03816,161.05965,147.04341,135.04344
26	Myristicin	C_11_H_12_O_3_	193.08592	193.08606	[M + H]^+^	5.55	1.28E+06	193.08583,165.05429,160.05147,135.04312,117.06930
27	Scopoletin	C_10_H_8_O_4_	193.04953	193.0497	[M + H]^+^	5.67	1.92E+06	178.02512,150.03026,137.05913,133.02785,122.03581
28	Umbelliferone	C_9_H_6_O_3_	163.03897	163.03903	[M + H]^+^	11.86	3.28E+06	163.03911,135.04419,121.02788,109.02848,95.04936
29	DL-Carvone	C_10_H_14_O	151.11174	151.11185	[M + H]^+^	5.82	1.42E+06	123.07984,109.06389,107.08508,95.08512,93.06918
30	Thymoxyacetic acid	C_12_H_16_O_3_	209.11722	209.11732	[M + H]^+^	6.01	2.12E+06	149.09509,133.10080,109.06394,105.06921
31	p-Cymene	C_10_H_14_	135.11683	135.11678	[M + H]^+^	6.29	1.70E+06	135.11687,93.06999
32	Perillic acid	C_10_H_14_O_2_	167.10666	167.10674	[M + H]^+^	6.62	1.81E+06	125.05868,121.10059,111.04363,109.06443,107.08519
33	4-Tert butylbenzoic acid	C_11_H_14_O_2_	179.10666	179.10671	[M + H]^+^	7.02	5.19E+06	133.10049,123.04314,119.08498,91.05397
34	Carvacrol	C_10_H_14_O	151.11174	151.11179	[M + H]^+^	7.52	9.02E+05	109.06396,105.06938,93.06905,91.05400
35	Nootkatone	C_15_H_22_O	219.17434	219.17434	[M + H]^+^	8.3	3.42E+06	201.16319,189.12659,177.12662,161.13178,149.13248,109.06479
36	Iso-E Super	C_16_H_26_O	235.20564	235.20541	[M + H]^+^	14.2	1.43E+05	191.17917,151.11130,147.11610,135.11632,123.11609
37	Oleanolic acid	C_30_H_48_O_3_	457.36762	457.36827	[M + H]^+^	20.33	4.94E+07	289.21579,275.19968,191.17848,181.12321,179.10584,163.14749
38	5,8-Dihydroxy-2,3,6-trimethoxy-1,4-naphthoquinone	C_13_H_12_O_7_	281.06558	281.0658	[M + H]^+^	3.31	1.90E+05	263.05228,235.05891,219.02913,139.03922
39	Hydroxyprogesterone	C_21_H_30_O_3_	331.22677	331.22673	[M + H]^+^	13.71	4.82E+06	331.22699,313.21619,295.20575,271.20587,191.14323
40	Epiallopregnanolone	C_21_H_34_O_3_	335.25807	335.25793	[M + H]^+^	14.84	1.88E+06	165.12691,159.11597,123.07957,121.10042,109.10036,107.08513
41	3-Hydroxycholest-5-en-7-one	C_27_H_44_O_2_	401.34141	401.34103	[M + H]^+^	24.54	4.71E+06	343.26334,341.24781,235.16939,221.15350,161.09619
42	Caffeic acid	C_9_H_8_O_4_	181.04953	181.04959	[M + H]^+^	2.88	2.05E+07	181.01306,163.08649,138.03114,125.05968
43	1-Salicylate glucuronide	C_13_H_14_O_9_	315.07106	315.07088	[M + H]^+^	3.39	1.20E+06	165.05453,153.05463,145.04943,143.03388
44	Gallic acid	C_7_H_6_O_5_	171.0288	171.02865	[M + H]^+^	3.87	5.60E+04	169.01334,125.02361,124.01605,123.00786,108.02087
45	3-Oxocyclopentanecarboxylic acid	C_6_H_8_O_3_	129.05462	129.05459	[M + H]^+^	4.19	2.13E+06	101.05936,83.04877,59.04896,55.05402
46	Ethyl gallate	C_9_H_10_O_5_	199.0601	199.06022	[M + H]^+^	5.29	2.10E+06	185.04442,171.02890,153.01829,127.03909,125.02340
47	3,4-Dimethoxybenzoic acid	C_9_H_10_O_4_	183.06518	183.0651	[M + H]^+^	5.64	2.41E+06	183.06540,167.03389,165.05461,151.03899,139.07542,137.02339
48	(±)-Abscisic acid	C_15_H_20_O_4_	265.14343	265.1433	[M + H]^+^	11.61	1.85E+06	249.14854,247.13275,233.15354,231.13814,223.13283
49	Monobutyl phthalate	C_12_H_14_O_4_	223.09648	223.09632	[M + H]^+^	11.87	1.18E+06	163.03863,149.02251,135.04327,131.08472,121.02756
50	Phenylbutyric acid	C_10_H_12_O_2_	165.09101	165.09096	[M + H]^+^	12.42	1.86E+07	147.08006,137.09532,121.10033,119.08482,103.05366
51	Mycophenolic acid	C_17_H_20_O_6_	321.13326	321.13301	[M + H]^+^	12.57	6.55E+05	285.11115,275.12698,109.06411
52	Ardisinol II	C_19_H_30_O_2_	291.23186	291.23205	[M + H]^+^	12.65	2.62E+06	178.08191,147.06284,119.08478,107.08492
53	Hexylresorcinol	C_12_H_18_O_2_	195.13796	195.13792	[M + H]^+^	13.3	2.72E+06	135.07948,109.02810,107.04833,95.04876
54	4-Heptylbenzoic acid	C_14_H_20_O_2_	221.15361	221.15354	[M + H]^+^	16.78	2.70E+06	177.16296,175.14723,147.11607,133.10062,121.10062,119.08498
55	9S,13R-12-Oxophytodienoic acid	C_18_H_28_O_3_	293.21112	293.21103	[M + H]^+^	17.49	1.23E+07	247.20465,173.13164,163.11095,161.13155,159.11589
56	Palmitoleic acid	C_16_H_30_O_2_	255.23186	255.23204	[M + H]^+^	18.8	1.91E+06	177.16295,149.13190,135.11616,125.09579,121.10053
57	Docosahexaenoic acid	C_22_H_32_O_2_	329.24751	329.2479	[M + H]^+^	19.72	1.54E+06	159.11644,133.10091,119.08460,111.07970,109.10036
58	Docosapentaenoic acid	C_22_H_34_O_2_	331.26316	331.26317	[M + H]^+^	20.66	3.33E+06	177.09004,159.11603,151.11090,135.11583,123.11612,109.10072
59	Ardisinol I	C_20_H_32_O_2_	305.24751	305.24746	[M + H]^+^	23.01	1.38E+07	287.23703,221.15367,207.13815,193.12265,179.10669
60	Tetradecanedioic acid	C_14_H_26_O_4_	259.19038	259.19058	[M + H]^+^	24.52	1.50E+06	241.17988,221.15350,209.15376,203.14274,193.12226,181.12217
61	Luteolin	C_15_H_10_O_6_	285.04046	285.04032	[M-H]^-^	10.72	1.96E+06	285.04047,257.04559,151.00322,133.02908
62	Embelin	C_17_H_26_O_4_	293.17583	293.17573	[M-H]^-^	14.2	2.97E+06	293.17584,265.18088,236.10515,221.15442,192.11511
63	Rapanone	C_19_H_30_O_4_	321.20713	321.20701	[M-H]^-^	23.19	2.47E+07	321.20758,293.21262,287.04538,275.20209,166.02695
65	Citric acid	C_6_H_8_O_7_	191.01972	191.01969	[M-H]^-^	2.06	1.64E+07	129.01854,103.03929,101.02355,87.00807,85.02887,71.01286
66	Methyl gallate	C_8_H_8_O_5_	183.0299	183.02989	[M-H]^-^	4.13	1.17E+06	183.02989,168.00621,154.02672,139.03993,134.03711,124.01635
67	Gentisic acid	C_7_H_6_O_4_	153.01933	153.01922	[M-H]^-^	4.62	3.77E+07	153.01840,109.02868,108.02099
68	Methyl salicylate	C_8_H_8_O_3_	151.04007	151.03973	[M-H]^-^	5.2	2.44E+06	135.00845,109.02922,91.08162
69	Salicylic acid	C_7_H_6_O_3_	137.02442	137.02438	[M-H]^-^	8.42	2.24E+07	121.02786,95.04888,93.03302
70	4-Oxododecanedioic acid	C_12_H_20_O_5_	243.1238	243.12383	[M-H]^-^	9.51	5.25E+06	243.12370,225.11298,207.10246,199.13362,183.10228,181.12299
71	3-Tert-butyladipic acid	C_10_H_18_O_4_	201.11323	201.11333	[M-H]^-^	10.81	8.14E+06	201.11299,183.10229,157.12300,139.11247,137.09686

**TABLE 2:** Details about 18 related metabolites of AJH serum components in ALI rats.

No.	Formula	Experimental MS (m/z)	Measured MS (m/z)	Ion mode	RT (min)	UPLC-Orbitrap Fusion MS/MS fragments (m/z)	Metabolic sequestration	Resource
1	C_16_H_12_O_6_	301.07066	301.07037	[M + H]^+^	13.37	286.04721,258.05237,167.03403	Methylation	Kaempferol
2	C_16_H_12_O_6_	299.05611	299.0559	[M-H]^-^	13.35	284.03262,212.04758,148.01617	Methylation	Luteolin
3	C_16_H_18_O_10_	371.09727	371.09769	[M + H]^+^	5.76	275.05511,247.06015,233.04451,205.04968	Methylation + glucuronide conjugation	Caffeic acid
4	C_15_H_16_O_10_	357.08162	357.08185	[M + H]^+^	4.56	261.03955,233.04457,219.02892,199.03398	Glucuronide conjugation	Caffeic acid
5	C_12_H_13_NO_3_	220.09682	220.09702	[M + H]^+^	9.23	159.11691,125.09621,107.08566,97.10132	Glycine conjugation	Methyl cinnamate
6	C_21_H_20_O_11_	449.10784	449.10804	[M + H]^+^	7.8	303.05026,257.04456,229.04973	Oxidation	Apigenin-7-O-glucoside
7	C_15_H_10_O_6_	287.05501	287.05489	[M + H]^+^	10.72	287.05493,259.08517,241.07463,153.01826	Hydroxylation + glucuronide conjugation	Apigenin-7-O-glucoside
8	C_21_H_22_O_11_	451.12349	451.12301	[M + H]^+^	8	259.06033,231.06540,195.02905,165.01845,153.01846	Hydration	Apigenin-7-O-glucoside
9	C_21_H_20_O_10_	433.11292	433.113	[M + H]^+^	9.86	329.06628,213.05504,153.01845	Deglucosylation	Luteolin-7-o-rutinoside
10	C_27_H_30_O_16_	611.16066	611.16063	[M + H]^+^	7.85	303.05011,177.05482,153.01840	Oxidation	Luteolin-7-o-rutinoside
11	C_28_H_32_O_16_	625.17631	625.17841	[M + H]^+^	8.94	317.06573,255.12033,207.06532,175.03914	Hydroxylation + methylation	Luteolin-7-o-rutinoside
12	C_27_H_30_O_15_	595.16575	595.16514	[M + H]^+^	8.79	303.05029,287.05542,153.05487	Oxidation	Apigenin-7-O-rutinoside
13	C_21_H_20_O_10_	433.11292	433.113	[M + H]^+^	9.86	287.05548,241.04991,185.05965,129.05496	Hydroxylation + glucuronide conjugation	Apigenin-7-O-rutinoside
14	C_28_H_32_O_16_	625.17631	625.17841	[M + H]^+^	8.94	317.06573,255.12033,207.06532,175.03914	Methylation	Apigenin-7-O-rutinoside
15	C_28_H_32_O_16_	625.17631	625.17841	[M + H]^+^	8.94	317.06573,255.12033,207.06532,175.03914	Methylation	Quercetin-3-O-rutinoside
16	C_21_H_20_O_11_	449.10784	449.10804	[M + H]^+^	7.8	345.06165,287.05524,247.06033,183.02893	Deglucosylation	Quercetin-3-O-rutinoside
17	C_26_H_43_NO_5_	448.30685	448.30862	[M-H]^-^	15.01	448.30679,404.31696,402.30160	Glycine conjugation	Deoxycholic acid
18	C_9_H_10_O_3_	165.05572	165.05534	[M-H]^-^	6.51	147.04488,119.04997,106.04213	Oxidation	Apigenin

### 3.3 Network analysis based on serum components in the ALI rat model

**FIGURE 4:**
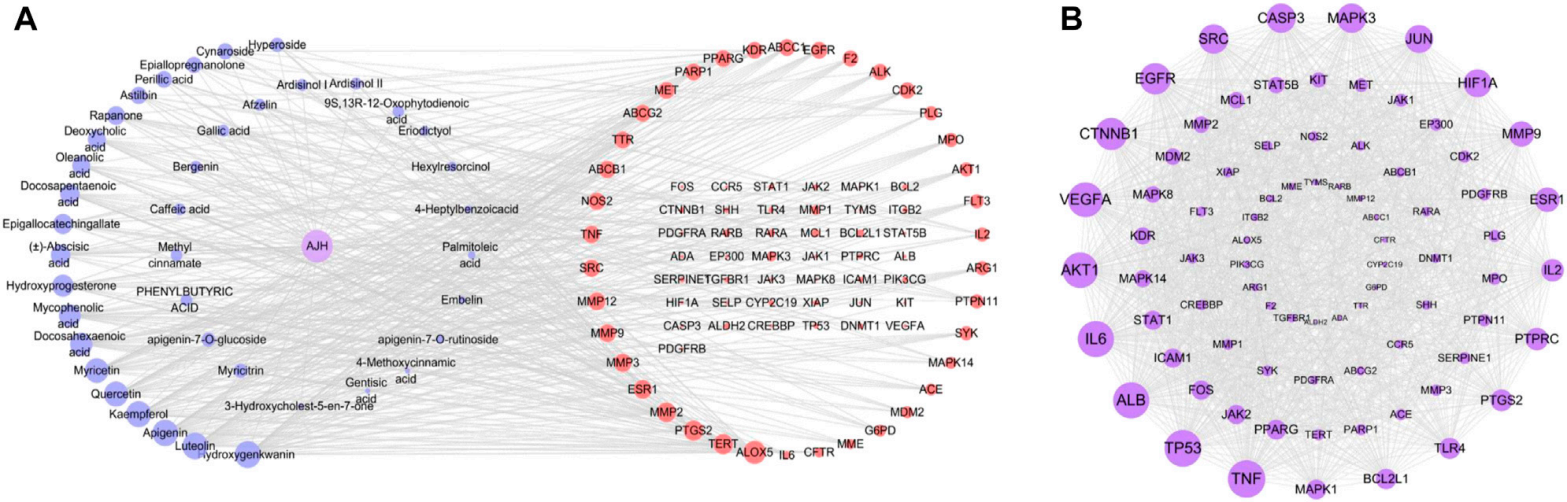
Network pharmacology prediction for AJH intervention in ALI. **(A)** AJH–serum component–target–ALI network. **(B)** PPI network.

In network analysis, we analyzed and selected 40 components among 71 serum components identified by UPLC-Orbitrap Fusion MS, which have acted on 81 overlapping targets. The AJH–serum component–target–ALI network (Figure 4A) included 122 nodes (one AJH, 40 serum components, and 81 targets) and 422 edges. The mean degree value of the components was 6.9, which indicated that the components regulated multiple targets to achieve therapeutic effects of AJH against ALI. In addition, hydroxygenkwanin, luteolin, apigenin, kaempferol, and quercetin acted on 28, 27, 27, 26, and 24 targets, respectively. Due to their important positions in this network, the aforementioned five components were selected as core components.

**FIGURE 5:**
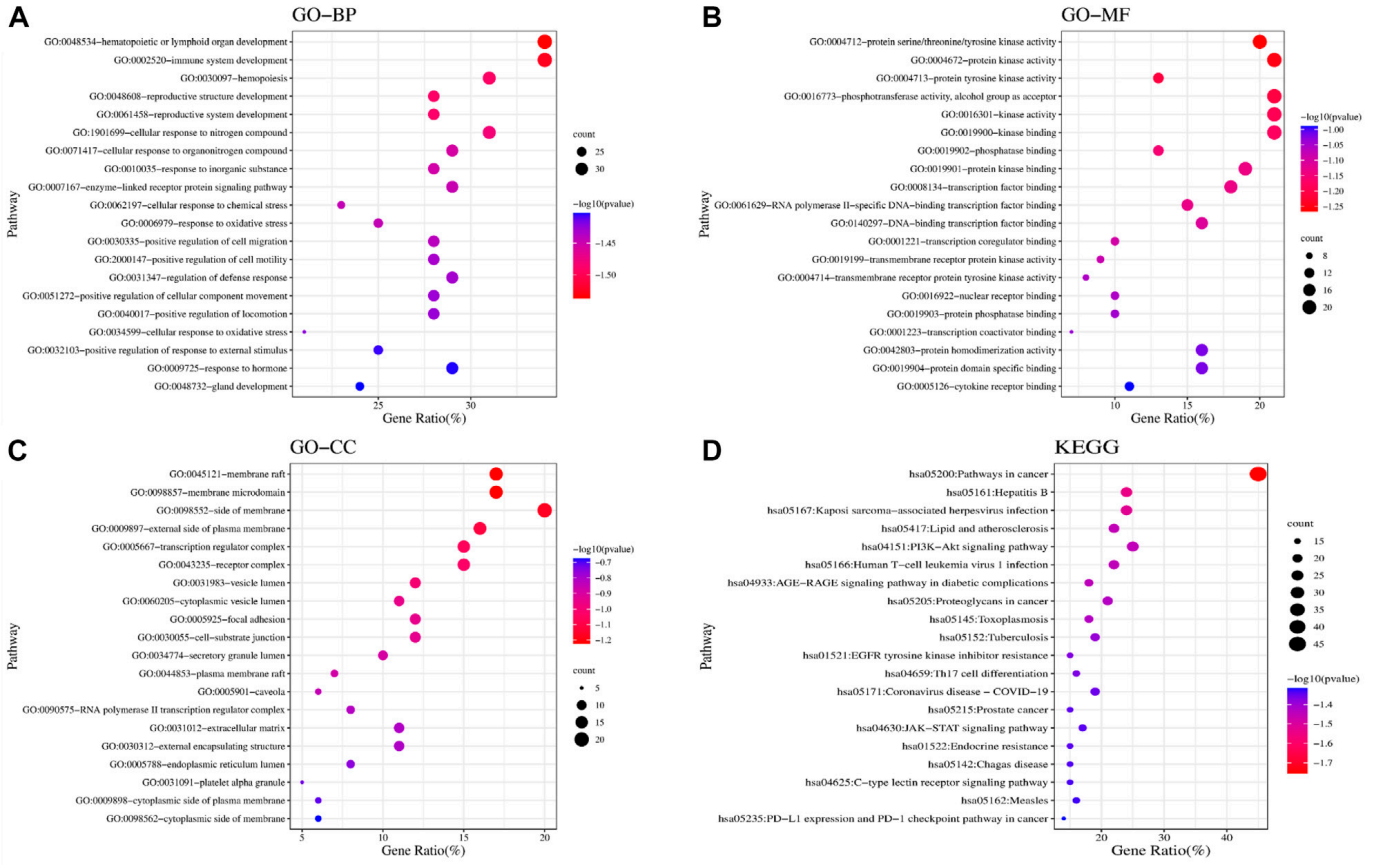
Results of GO and KEGG enrichment analyses. Bubble charts of **(A)** GOBP; **(B)** GOMF; **(C)** GOCC; and **(D)** KEGG enrichment analysis.

We found 81 overlapping targets to construct the PPI network (Figure 4B). The average node degree was 34.5, in which 36 targets (degree >34.5) were selected as core targets. The core targets with topological significance, including TNF, tumor protein (TP) 53, albumin (ALB), IL-6, AKT serine/threonine kinase (AKT)1, vascular endothelial growth factor A (VEGFA), epidermal growth factor receptor (EGFR), mitogen-activated protein kinase (MAPK), and toll-like receptor (TLR) 4, might play an important role in the molecular mechanism of AJH against ALI. The number of Gene Ontology (GO) biological processes (BP), GO cellular components (CC), and GO molecular functions (MF) was 1,499, 62, and 108, respectively. GOBP, GOCC, and GOMF with all top 20 *p*-values were screened and represented by a graphical bubble with the *p*-value, as shown in Figures 5A–C and Supplementary Table S4. Finally, we found 171 related signaling pathways. The top 20 *p*-values were represented by a graphical bubble, as shown in Figure 5D and Supplementary Table S5, which included the PI3K-Akt, AGE-RAGE, and JAK-STAT signaling pathways.

### 3.4 Metabolomics analysis

**FIGURE 6:**
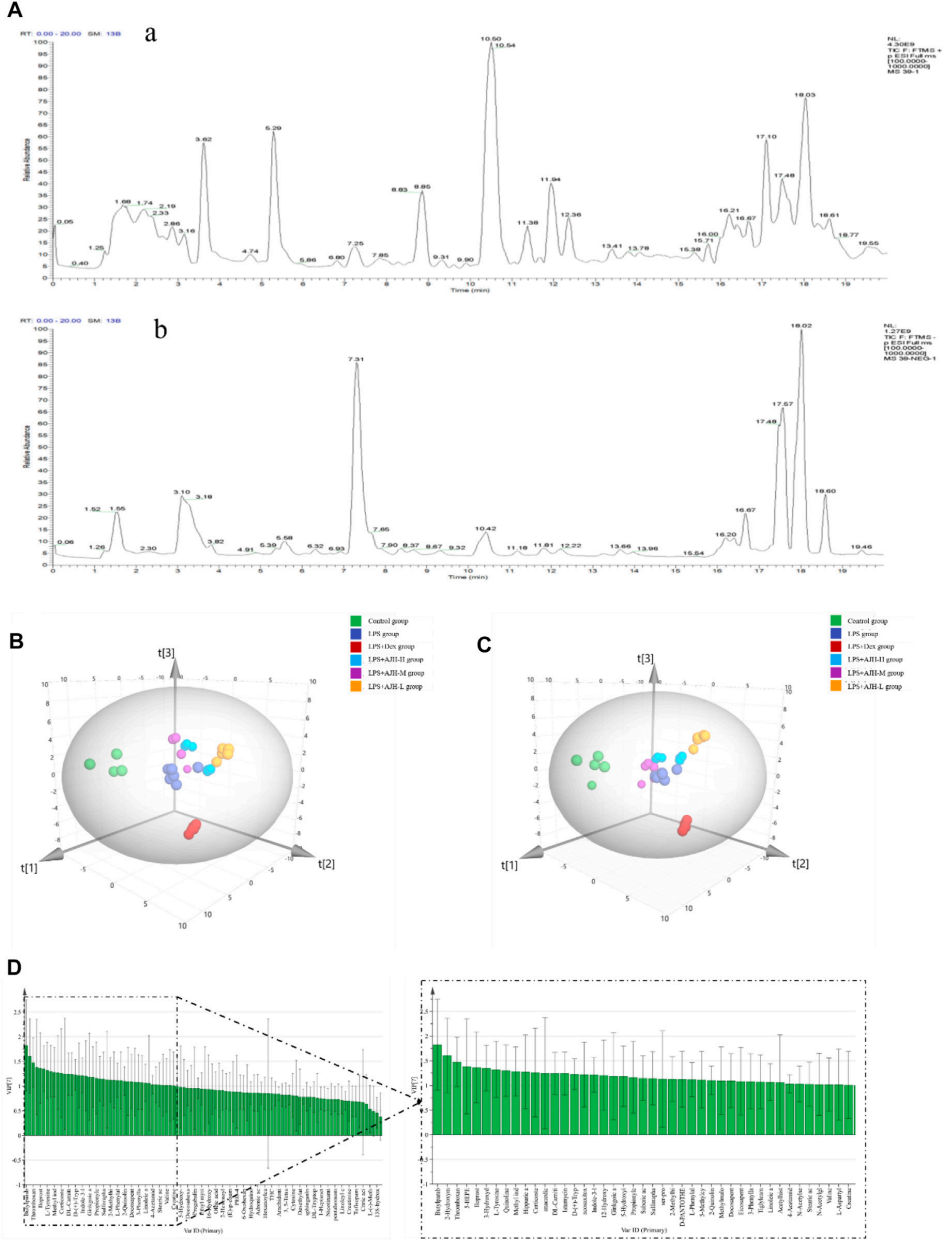
Metabolomics analysis based on the multivariate statistical method. **(A)** Total ion chromatograms (TICs) of metabolic samples (a. positive ion mode; b. negative ion mode); **(B)** 3D PCA score plot; **(C)** 3D PLS-DA score plot; **(D)** VIP chart.

According to the established UPLC-Orbitrap Fusion MS analysis method, the serum sample data of the control, LPS, LPS + Dex, LPS + AJH-L, LPS + AJH-M, and LPS + AJH-H groups were analyzed, and the TIC spectra are shown in Figure 6A with a total of 102 metabolites annotated and identified (Table 3). PCA can reduce the dimensionality of complex datasets to form several main components, reflect the natural distribution of different groups, and find outliers (Huang et al., 2018). The data of 102 metabolites in each group were performed using SIMCA 14.1 software to establish the PCA score plot (Figure 6B). The control, LPS, LPS + Dex, and LPS + AJH-treated groups were significantly scattered and clustered. In addition, the control group was significantly separated from the LPS group, which further proved that the ALI rat model was successfully replicated, as shown in Figure 6B. Compared with the control group and LPS group, the LPS + Dex and LPS + AJH-treated groups are close to the control group and away from the LPS group. The aforementioned PCA results illustrated the effectiveness of AJH in the intervention of ALI.

**TABLE 3:** Metabolites in serum samples.

No.	Rt (min)	Observed (m/z)	Ion mode	Identification	Formula	Tendency in model samples *versus* control samples	VIP
1	1.21	145.1579	[M + H]^+^	Spermidine	C_7_H_19_N_3_	↓	0.9996
2	1.43	103.0999	[M + H]^+^	Choline	C_5_H_13_NO	↓	0.7434
3	1.58	131.0695	[M + H]^+^	Creatine	C_4_H_9_N_3_O_2_	↑	1.0110
4	1.58	113.0591	[M + H]^+^	Creatinine	C_4_H_7_N_3_O	↓	0.6972
5	1.58	161.1053	[M + H]^+^	DL-Carnitine	C_7_H_15_NO_3_	↑	1.2442
6	1.59	111.0434	[M + H]^+^	Cytosine	C_4_H_5_N_3_O	↓	0.8123
7	1.59	159.1259	[M + H]^+^	Pregabalin	C_8_H_17_NO_2_	↓	0.9559
8	1.59	117.0790	[M + H]^+^	Valine	C_5_H_11_NO_2_	↑	1.0165
9	1.60	261.1211	[M + H]^+^	Lotaustralin	C_11_H_19_NO_6_	↑	0.9396
10	1.67	153.0900	[M + H]^+^	Acetylhistamine	C_7_H_11_N_3_O	↑	1.0611
11	1.75	149.0510	[M + H]^+^	L-(−)-Methionine	C_5_H_11_NO_2_S	↓	0.5381
12	1.88	122.0480	[M + H]^+^	Nicotinamide	C_6_H_6_N_2_O	↓	0.7372
13	1.88	168.0283	[M + H]^+^	Uric acid	C_5_H_4_N_4_O_3_	↓	0.8517
14	1.89	257.1010	[M + H]^+^	5-Methylcytidine	C_10_H_15_N_3_O_5_	↑	0.8651
15	1.89	241.1062	[M + H]^+^	5-Methyldeoxycytidine	C_10_H_15_N_3_O_4_	↑	0.8870
16	2.09	125.0590	[M + H]^+^	5-Methylcytosine	C_5_H_7_N_3_O	↑	1.1157
17	2.09	181.0739	[M + H]^+^	L-Tyrosine	C_9_H_11_NO_3_	↑	1.3252
18	2.15	118.0418	[M + H]^+^	Coumarone	C_8_H_6_O	↓	0.9755
19	2.22	132.0975	[M + H]^+^	1-Heptanethiol	C_7_H_16_S	↓	0.7524
20	2.23	131.0946	[M + H]^+^	L-Isoleucine	C_6_H_13_NO_2_	↓	0.8602
21	2.44	164.0472	[M + H]^+^	(E)-p-Coumaric acid	C_9_H_8_O_3_	↑	0.8918
22	2.68	217.1314	[M + H]^+^	Propionylcarnitine	C_10_H_19_NO_4_	↑	1.1635
23	3.10	174.0793	[M + H]^+^	Edaravone	C_10_H_10_N_2_O	↑	0.4934
24	3.18	173.1051	[M + H]^+^	N-Acetyl-L-leucine	C_8_H_15_NO_3_	↓	0.7597
25	3.18	315.1291	[M + H]^+^	Neosaxitoxin	C_10_H_17_N_7_O_5_	↑	1.2204
26	3.39	191.0582	[M + H]^+^	5-Hydroxyindole-3-acetic acid	C_10_H_9_NO_3_	↑	1.1850
27	3.48	126.0428	[M + H]^+^	Thymine	C_5_H_6_N_2_O_2_	↓	0.8188
28	3.50	168.0875	[M + H]^+^	Pyridoxamine	C_8_H_12_N_2_O_2_	↓	0.9556
29	3.59	165.0789	[M + H]^+^	L-Phenylalanine	C_9_H_11_NO_2_	↑	1.1184
30	3.87	194.1153	[M + H]^+^	PEG-4	C_8_H_18_O_5_	↑	0.8846
31	3.98	219.1106	[M + H]^+^	D-Pantothenic acid	C_9_H_17_NO_5_	↑	1.1255
32	5.13	243.1469	[M + H]^+^	Tiglylcarnitine	C_12_H_21_NO_4_	↑	1.0748
33	5.28	187.0607	[M + H]^+^	Desethylatrazine	C_6_H_10_ClN_5_	↓	0.7858
34	5.29	220.0846	[M + H]^+^	5-Hydroxy-DL-tryptophan	C_11_H_12_N_2_O_3_	↓	0.9817
35	5.29	204.0899	[M + H]^+^	DL-Tryptophan	C_11_H_12_N_2_O_2_	↓	0.7795
36	5.80	262.1314	[M + H]^+^	Methohexital	C_14_H_18_N_2_O_3_	↓	0.7853
37	5.80	382.1966	[M + H]^+^	Tofisopam	C_22_H_26_N_2_O_4_	↑	0.6902
38	5.95	173.0476	[M + H]^+^	2-Quinolinecarboxylic acid	C_10_H_7_NO_2_	↓	1.1023
39	5.95	179.0582	[M + H]^+^	Hippuric acid	C_9_H_9_NO_3_	↓	1.2751
40	6.05	260.1370	[M + H]^+^	L-gamma-Glutamyl-L-leucine	C_11_H_20_N_2_O_5_	↓	0.7021
41	6.05	218.1055	[M + H]^+^	N-Acetylserotonin	C_12_H_14_N_2_O_2_	↑	1.0294
42	6.53	257.1625	[M + H]^+^	2-Hexenoylcarnitine	C_13_H_23_NO_4_	↓	0.9174
43	6.56	189.0425	[M + H]^+^	Kynurenic acid	C_10_H_7_NO_3_	↓	0.8463
44	6.59	294.1213	[M + H]^+^	Aspartame	C_14_H_18_N_2_O_5_	↓	0.7352
45	8.52	259.1782	[M + H]^+^	Hexanoylcarnitine	C_13_H_25_NO_4_	↓	0.8575
46	8.54	177.0281	[M + H]^+^	Sulforaphane	C_6_H_11_NOS_2_	↑	1.1457
47	9.32	205.0738	[M + H]^+^	Indole-3-lactic acid	C_11_H_11_NO_3_	↑	1.2161
48	10.41	337.1861	[M + H]^+^	Istamycin AO	C_13_H_27_N_3_O_7_	↑	1.2429
49	12.85	219.1292	[M + H]^+^	Pentahomomethionine	C_10_H_21_NO_2_S	↓	0.7331
50	13.01	129.0579	[M + H]^+^	Quinoline	C_9_H_7_N	↑	1.3006
51	15.74	313.2253	[M + H]^+^	9-Decenoylcarnitine	C_17_H_31_NO_4_	↓	0.7838
52	15.90	346.2144	[M + H]^+^	Corticosterone	C_21_H_30_O_4_	↑	1.2626
53	15.95	341.2564	[M + H]^+^	Trans-2-Dodecenoylcarnitine	C_19_H_35_NO_4_	↓	0.8213
54	16.00	367.2721	[M + H]^+^	3, 5-Tetradecadiencarnitine	C_21_H_37_NO_4_	↓	0.8208
55	16.04	210.1232	[M + H]^+^	Jasmonic acid	C_12_H_18_O_3_	↑	0.9176
56	16.04	301.2979	[M + H]^+^	Sphinganine	C_18_H_39_NO_2_	↓	0.7849
57	16.13	360.2298	[M + H]^+^	Iloprost	C_22_H_32_O_4_	↑	1.3640
58	16.18	423.3347	[M + H]^+^	Linoleyl carnitine	C_25_H_45_NO_4_	↓	0.7149
59	16.24	399.3347	[M + H]^+^	Palmitoylcarnitine	C_23_H_45_NO_4_	↓	0.7318
60	16.45	314.2219	[M + H]^+^	THC	C_21_H_30_O_2_	↑	0.8493
61	16.58	262.1568	[M + H]^+^	3″-Hydroxy-geranylhydroquinone	C_16_H_22_O_3_	↑	0.9573
62	16.69	308.2326	[M + H]^+^	Eicosapentaenoic acid	C_20_H_30_O_2_	↑	1.0847
63	16.70	304.2399	[M + H]^+^	Arachidonic acid	C_20_H_32_O_2_	↓	0.8450
64	18.04	110.0368	[M + H]^+^	Hydroquinone	C_6_H_6_O_2_	↓	0.8630
65	13.02	189.0789	[M + H]^+^	Methyl indole-3-acetate	C_11_H_11_NO_2_	↑	1.2827
66	1.60	117.0420	[M-H]^-^	N-Acetylglycine	C_4_H_7_NO_3_	↑	1.0214
67	1.67	117.0784	[M-H]^-^	5-Aminovaleric acid	C_5_H_11_NO_2_	↓	0.9335
68	1.78	118.0261	[M-H]^-^	Methylmalonic acid	C_4_H_6_O_4_	↑	1.0963
69	1.78	202.0949	[M-H]^-^	Ser-pro	C_8_H_14_N_2_O_4_	↑	1.1308
70	2.02	129.0420	[M-H]^-^	4-Oxoproline	C_5_H_7_NO_3_	↑	0.6355
71	2.06	192.0262	[M-H]^-^	Citric acid	C_6_H_8_O_7_	↑	0.6741
72	2.82	183.0525	[M-H]^-^	4-Pyridoxic acid	C_8_H_9_NO_4_	↓	0.3797
73	2.84	116.0469	[M-H]^-^	Methyl acetoacetate	C_5_H_8_O_3_	↓	0.6839
74	4.68	145.0732	[M-H]^-^	4-Acetamidobutanoic acid	C_6_H_11_NO_3_	↑	1.0323
75	5.24	204.0895	[M-H]^-^	D-(+)-Tryptophan	C_11_H_12_N_2_O_2_	↓	1.2278
76	5.39	214.0117	[M-H]^-^	L-Aspartyl-4-phosphate	C_4_H_9_NO_7_P	↓	1.0156
77	5.98	260.1371	[M-H]^-^	Leu-Glu	C_11_H_20_N_2_O_5_	↓	0.6926
78	6.14	130.0624	[M-H]^-^	6-Oxohexanoic acid	C_6_H_10_O_3_	↓	0.8673
79	8.57	193.0733	[M-H]^-^	2-Methylhippuric acid	C_10_H_11_NO_3_	↑	1.1274
80	8.70	166.0623	[M-H]^-^	3-Phenyllactic acid	C_9_H_10_O_3_	↑	1.0823
81	9.84	174.0885	[M-H]^-^	Suberic acid	C_8_H_14_O_4_	↓	1.1495
82	16.00	194.0936	[M-H]^-^	Butylparaben	C_11_H_14_O_3_	↑	1.8263
83	16.01	370.2355	[M-H]^-^	Thromboxane B2	C_20_H_34_O_6_	↑	1.4778
84	16.14	188.1405	[M-H]^-^	3-Hydroxydecanoic acid	C_10_H_20_O_3_	↑	1.3511
85	16.37	244.2034	[M-H]^-^	2-Hydroxymyristic acid	C_14_H_28_O_3_	↑	1.6081
86	16.46	216.1719	[M-H]^-^	12-Hydroxylauric acid	C_12_H_24_O_3_	↑	1.2065
87	16.56	318.2192	[M-H]^-^	5-HEPE	C_20_H_30_O_3_	↑	1.3855
88	16.61	242.1877	[M-H]^-^	3-Oxotetradecanoic acid	C_14_H_26_O_3_	↓	0.4613
89	16.61	296.2350	[M-H]^-^	13S-Hydroxyoctadecadienoic acid	C_18_H_32_O_3_	↓	0.8055
90	16.63	272.2349	[M-H]^-^	16-Hydroxyhexadecanoic acid	C_16_H_32_O_3_	↓	0.9383
91	16.75	346.2504	[M-H]^-^	Ginkgoic acid	C_22_H_34_O_3_	↑	1.1884
92	16.88	348.2662	[M-H]^-^	Anacardic acid	C_22_H_36_O_3_	↑	1.2482
93	17.44	328.2406	[M-H]^-^	Docosahexaenoic acid	C_22_H_32_O_2_	↓	0.9610
94	17.61	280.2402	[M-H]^-^	Linoleic acid	C_18_H_32_O_2_	↑	1.0674
95	17.62	330.2553	[M-H]^-^	Docosapentaenoic acid	C_22_H_34_O_2_	↑	1.0961
96	17.86	306.2554	[M-H]^-^	8Z,11Z,14Z-Eicosatrienoic acid	C_20_H_34_O_2_	↓	0.8787
97	17.88	256.2402	[M-H]^-^	Ethyl myristate	C_16_H_32_O_2_	↓	0.9412
98	17.99	332.2711	[M-H]^-^	Adrenic acid	C_22_H_36_O_2_	↓	0.8615
99	18.01	282.2558	[M-H]^-^	Oleic acid	C_18_H_34_O_2_	↓	0.9214
100	18.58	284.2717	[M-H]^-^	Stearic acid	C_18_H_36_O_2_	↓	1.0277
11	18.64	310.2870	[M-H]^-^	11(Z)-Eicosenoic acid	C_20_H_38_O_2_	↓	0.8998
102	19.37	312.3023	[M-H]^-^	Arachidic acid	C_20_H_40_O_2_	↓	0.8624

In order to further select potential biomarkers and determine the difference between the control group and LPS group, between control, LPS groups, and LPS + AJH-treated groups, and between control, LPS groups, and LPS + Dex group, the metabolomics data were placed in a supervised method of PLS-DA (Figure 6C). The R^2^X, R^2^Y, and Q^2^ in the PLS-DA model were 0.782, 0.964, and 0.916, respectively (Q^2^ > 0.5). After setting permutations at 200 times, the permutation test of the PLS-DA model was applied to further assess the reliability level. As shown in Supplementary Figure S1, all the Q^2^ and *R* ^2^ values of Y-permuted models were lower than those of the original models, which showed that the PLS-DA model has a significant explanation or prediction ability and is not over-fitted. Based on VIP >1.0 (Figure 6D), 43 components were selected as potential biomarkers and are presented in Table 3.

**FIGURE 7:**
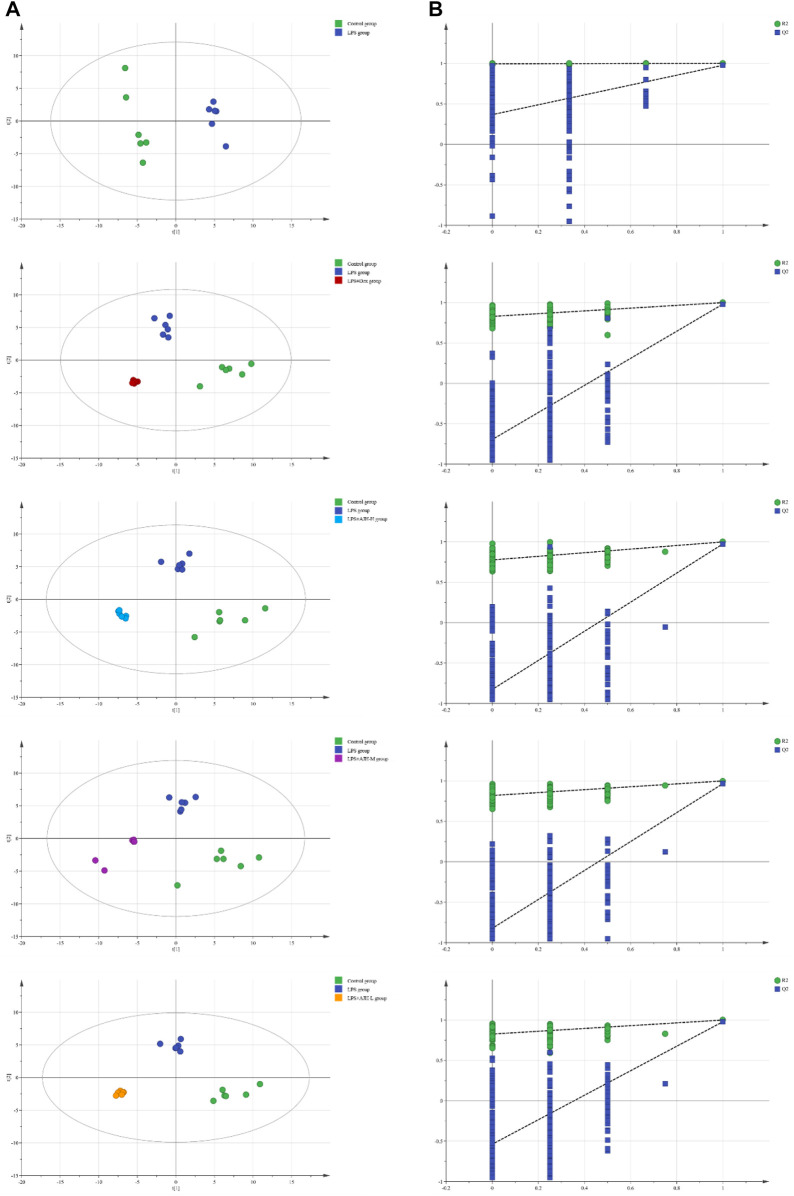
Metabolomics analysis between control and LPS groups, between control, LPS, and LPS + Dex groups, between control, LPS, and LPS + AJH-H groups, between control, LPS, and LPS + AJH-M groups, and between control, LPS, and LPS + AJH-L groups. **(A)** PLS-DA score plot; **(B)** permutation test of the PLS-DA model.

**FIGURE 8:**
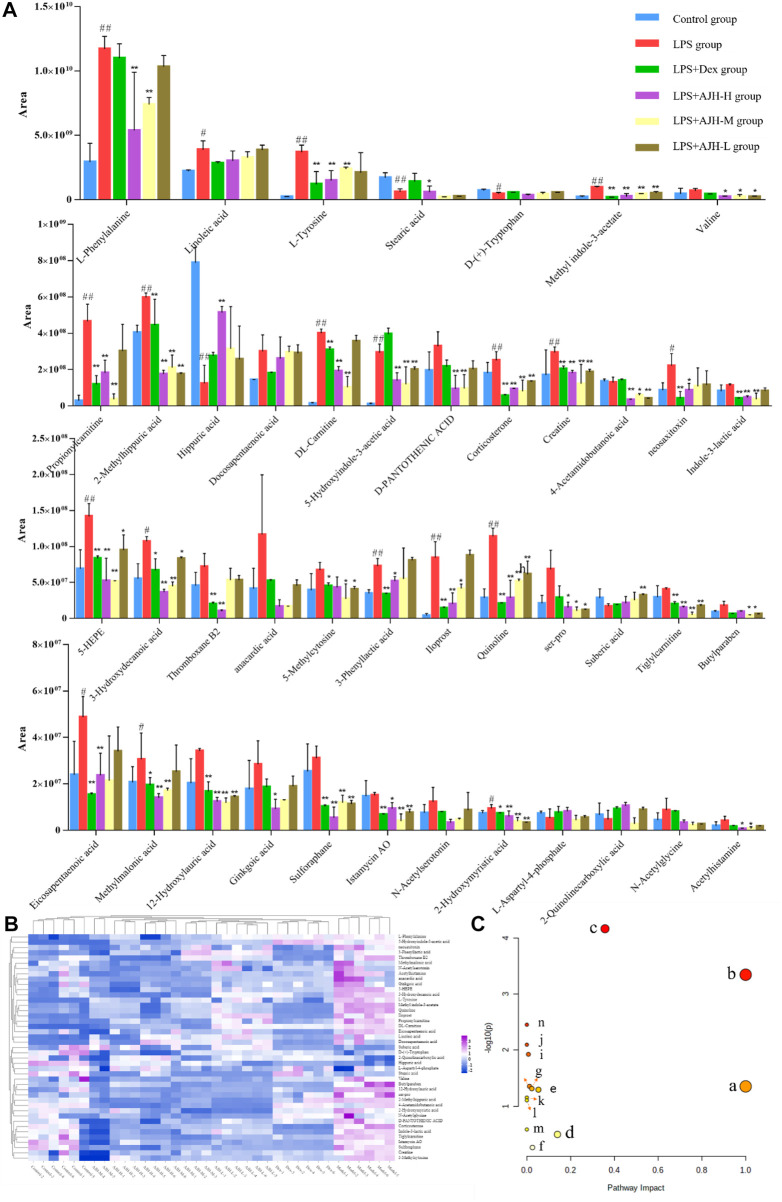
Systems analysis of potential metabolomic biomarkers in each group. **(A)** Histogram of the relative content of potential metabolomic biomarkers (^#^ *p* < 0.05, ^##^ *p* < 0.01 vs the control group; *p < 0.05, **p < 0.01 vs the LPS group). **(B)** Heatmap of potential metabolomic biomarkers in control, LPS, LPS + Dex, LPS + AJH-H, LPS + AJH-M, and LPS + AJH-L groups. **(C)** Metabolic pathway analysis associated with the pathogenesis of ALI using MetaboAnalyst 5.0 (a. linoleic acid metabolism; b. phenylalanine, tyrosine, and tryptophan biosynthesis; c. phenylalanine metabolism; d. tyrosine metabolism; e. tryptophan metabolism; f. steroid hormone biosynthesis; g. valine, leucine, and isoleucine degradation; h. arginine and proline metabolism; i. pantothenate and CoA biosynthesis; j. aminoacyl-tRNA biosynthesis; k. valine, leucine, and isoleucine biosynthesis; l. ubiquinone and other terpenoid–quinone biosynthesis; m. glycine, serine, and threonine metabolism; n. biosynthesis of unsaturated fatty acids).

In Figure 7A, the control group and LPS group were scattered and clustered, which showed that the ALI rat model was successfully established. Then, the control, LPS, and LPS + Dex/AJH-H/AJH-M/AJH-L groups were obviously clustered into three individual groups and separated from each other (Figure 7A), and the results of the permutation test indicated that the PLS-DA model was reliable and not over-fitted (Figure 7B). According to the cleavage law of mass spectrometry, HCDM, Compound Discoverer database, and references, we identified 43 potential biomarkers. The further analysis was aimed at the content level and tendency of the identified biomarkers in each group, as shown in Figures 8A, B. Compared with the control group, the levels of L-phenylalanine, linoleic acid, L-tyrosine, methyl indole-3-acetate, propionylcarnitine, 2-methylhippuric acid, DL-carnitine, corticosterone, creatine, neosaxitoxin, 5-HEPE, 3-hydroxydecanoic acid, 3-phenyllactic acid, iloprost, quinoline, eicosapentaenoic acid, methylmalonic acid, and 2-hydroxymyristic acid were significantly upregulated in the LPS group (*p* < 0.05/*p* < 0.01). The levels of stearic acid, D-(+)-tryptophan, and hippuric acid were downregulated in the LPS group (*p* < 0.05/*p* < 0.01). We found that the LPS + AJH-treated groups had a callback effect on the aforementioned potential biomarkers (*p* < 0.05 and *p* < 0.01). Once again, the therapeutic effects of LPS + AJH-M and LPS + AJH-H groups were better than those of the LPS + AJH-L group.

To further classify the metabolic pathways related to ALI, the 43 identified metabolic biomarkers were introduced into MetaboAnalyst 5.0 software to obtain 14 metabolic pathways, as shown in Figure 8C and Table 4. Based on the screening criteria of the pathway impact value greater than 0.1 and literature reports (Hu et al., 2020), four metabolic pathways closely associated with AJH intervention in ALI were obtained: linoleic acid metabolism, phenylalanine, tyrosine, and tryptophan biosynthesis, phenylalanine metabolism, and tyrosine metabolism.

**TABLE 4:** Details about 14 metabolic pathways.

No.	Pathway name	Match status	P	-log(p)	Holmp	Impact	Biomarkers
a	Linoleic acid metabolism	1/5	0.044409	1.3525	1	1	Linoleate
b	Phenylalanine, tyrosine, and tryptophan biosynthesis	2/4	0.0004501	3.3467	0.03736	1	L-Phenylalanine and L-tyrosine
c	Phenylalanine metabolism	3/10	0.0000679	4.168	0.00571	0.35714	L-Tyrosine, hippurate, and L-phenylalanine
d	Tyrosine metabolism	1/42	0.32039	0.49432	1	0.13972	L-Tyrosine
e	Tryptophan metabolism	2/41	0.050822	1.2939	1	0.05291	N-Acetylserotonin and 5-hydroxyindoleacetate
f	Steroid hormone biosynthesis	1/85	0.54752	0.2616	1	0.02562	Corticosterone
g	Valine, leucine, and isoleucine degradation	2/40	0.048593	1.3134	1	0.02264	L-Valine and methylmalonate
h	Arginine and proline metabolism	2/38	0.044248	1.3541	1	0.01212	Creatine and 4-acetamidobutanoate
i	Pantothenate and CoA biosynthesis	2/19	0.011871	1.9255	0.94967	0.00714	Pantothenate and L-valine
j	Aminoacyl-tRNA biosynthesis	3/48	0.0079837	2.0978	0.64668	0	L-Phenylalanine, L-valine, and L-tyrosine
k	Valine, leucine, and isoleucine biosynthesis	1/8	0.070168	1.1539	1	0	L-Valine
l	Ubiquinone and other terpenoid–quinone biosynthesis	1/9	0.07861	1.1045	1	0	L-Tyrosine
m	Glycine, serine, and threonine metabolism	1/33	0.26108	0.58322	1	0	Creatine
n	Biosynthesis of unsaturated fatty acids	3/36	0.0035154	2.454	0.28826	0	Octadecanoic acid, linoleate, and (5Z,8Z,11Z,14Z,17Z)-icosapentaenoic acid

## 4 Discussion

ALI is caused by the excessive release of the inflammatory cytokines to break the balance of inflammatory and anti-inflammatory cytokines, which can further develop into the systemic inflammatory response syndrome (SIRS), acute respiratory distress syndrome (ARDS), and eventually to multiple organ failure (MOF) (Liu and Chen, 2016). Therefore, inflammation is of great significance in ALI, especially chronic obstructive pulmonary disease and acute exacerbation of chronic obstructive pulmonary disease. Inflammation causes the infiltration of inflammatory cells and can release the chemokines and pro-inflammatory cytokines (Han et al., 2022; Yuan et al., 2022). The related study has demonstrated that TNF-α, IL-1β, IL-6, IL-8, and IL-18 are most closely associated with the outcome of ALI and are the current diagnosis and prognosis method (Parsons et al., 2005). Lv et al. (2021) indicated that fluorofenidone has a therapeutic effect on ALI by alleviating the lung tissue structure and decreasing the levels of IL-1β, IL-6, and TNF-α in the BALF. In our study, the level of IL-6 and IL-10 in serum and BALF were significantly upregulated or downregulated in ALI model rats compared with the control group, which might imply that AJH had anti-inflammatory capability. In addition, the lung wet/dry (W/D) ratio and indexes of the thymus and spleen in the LPS group were markedly elevated. The aforementioned indexes were also improved in the AJH-treated groups at different levels. Therefore, our study proved that AJH improved lung function and decreased systemic inflammation and provided evidence of an anti-inflammatory role of AJH at multiple levels. In our histopathological study, marked inflammatory cell infiltration, alveolar ectasia and fusion, and bronchiolar stenosis were improved in the AJH-treated groups at different levels.

In our previous study, we identified 236 components in AJH. Network analysis indicated that 41 core components could regulate the inflammation-related pathways, and the core components have anti-inflammatory effects (Feng et al., 2022). According to Bencao Shiyi, AJH is effective in reducing swelling, resolving phlegm, and relieving cough, which is widely used for treating cough, wheezing, blood in sputum, chronic bronchitis, and damp heat jaundice. Zhou et al. (2012) investigated and found that AJH is effective in preventing respiratory diseases, which can relieve symptoms and improve the pulmonary function in chronic obstructive pulmonary disease. Recently, network analysis illustrates the complex interactions among the biological systems, drugs, and disease from a network perspective, which opens avenues for new research ideas and technical means to study the action mechanisms of the CM formulae (Li, 2021). In our study, we further identified the serum components in ALI rats and used integrated network analysis and metabolomics to study the therapeutic effects of AJH in the ALI treatment. We identified a total of 71 serum components and 18 related metabolites in the ALI rat model, mainly including flavonoids, phenylpropanoids, and terpenes.

Because serum components in AJH might act on the diverse potential targets, we collected targets of serum components and targets associated with ALI to further explore the mechanisms underlying AJH against ALI by network analysis. According to degree values in the AJH–component–target–ALI network, we selected the five core serum components, including hydroxygenkwanin, luteolin, apigenin, kaempferol, and quercetin. The aforementioned core serum components of AJH belonged to flavonoids. Flavonoids are ubiquitous in all vascular plants and have been recognized to possess anti-inflammatory, anti-atherogenic, anti-allergic, and anti-cancer activities *in vitro* and *in vivo* (Ana et al., 2008; Chagas et al., 2022). Jiang et al. (2014) indicated that hydroxygenkwanin, luteolin, and apigenin possess immunoregulatory functions in LPS-activated RAW264.7 cells by suppressing the production of NO, which shows significant anti-inflammatory and antioxidant activities. In our previous study, luteolin, kaempferol, and quercetin showed an anti-inflammatory effect by regulating the IL-6 and MMP9 levels in the TNF-α-induced A549 cell model (Han et al., 2021; Feng et al., 2022). In addition, the PPI network was constructed to select the key targets among 81 overlapping targets, including TNF, TP53, ALB, IL-6, AKT1, VEGFA, EGFR, MAPK, and TLR4. The related studies revealed that IL-6, VEGFA, EGFR, and MAPK are linked to inflammatory pathogenesis of ALI (Chu et al., 2016; Ying et al., 2022). These key targets might be regulated to achieve anti-inflammatory activity of AJH against ALI. According to GO and KEGG analysis, some targets among the 81 overlapping targets are highly enriched inflammatory-related pathways, including the PI3K-Akt, AGE-RAGE, and JAK-STAT signaling pathways. Yu et al. (2022) suggested that the AGE-RAGE signaling pathway is activated to further increase the levels of pro-inflammatory cytokines, including IL-1β and TNF. The relevant studies illustrated that the PI3K-AKT could regulate downstream inflammatory cytokines, which plays a crucial role in inflammatory response (Schaper and Rose-John, 2015; Nazanin et al., 2020). Therefore, the serum components of AJH might act on 81 overlapping targets to regulate inflammatory-related pathways.

The metabolomics analysis revealed some potential biomarkers related with the therapeutic effects of AJH for LPS-induced ALI. Upregulation of L-Phenylalanine and downregulation of L-tyrosine in the ALI rat model were the intermediates of phenylalanine, tyrosine, and tryptophan biosynthesis. Capuron et al. (2011) illustrated that increased inflammation is linked to reduced tryptophan levels to improve tryptophan catabolism, and the inflammation was associated with increasing phenylalanine levels at the expense of tyrosine. We found that L-phenylalanine and L-tyrosine concentrations were called back in the LPS + AJH-treated groups, indicating the therapeutic effects of AJH on LPS-induced ALI rats. In addition, due to mitochondria being vulnerable to oxidation stress and pro-inflammatory mediators (Liu and Chen, 2016). Ji (2020), a LPS-induced macrophage inflammatory environment reduces the core enzyme levels of mitochondria and affects mitochondrial function that is closely related to the stability of the citrate cycle. In our study, the creatine levels associated with the citrate cycle were significantly downregulated in the LPS + AJH-treated groups, which revealed that AJH could protect LPS-induced ALI rats by restoring the disordered metabolism. Furthermore, the relative study suggested that fatty acids and derivatives play essential roles in various physiological processes, which have endogenous anti-inflammatory, antibacterial, antifungal, antiviral, and immunomodulatory agents (Das, 2018) and were considered clinical diagnosis indexes associated with inflammation (Does et al., 2018). Linoleic acid, as typical essential fatty acids, is a precursor and generates dihomo-gamma-linolenic acid and arachidonic acid (Evans et al., 2014). According to our results, the levels of linoleic acid in ALI rats were increased, but its concentration was called back in the LPS + AJH-treated groups. The previous study showed that arachidonic acid, as an important precursor of inflammatory mediators (eicosanoids), can regulate inflammatory and immune responses (Chandrasekharan et al., 2016). Meanwhile, arachidonic acid was related to inflammatory pathways including TLR4 and MAPK (Mateu et al., 2016), which could be evidenced from our network analysis results. Moreover, the level of arachidonic acid among 102 metabolites was decreased in ALI rats in our study, which may be converted into pro-inflammatory products and is consistent with the previous results (Wang et al., 2020).

Therefore, AJH modulation on potential biomarker metabolism might be related to the regulation of inflammatory-related pathways selected by network analysis of serum components. In addition, we further hypothesized that AJH exerts anti-inflammatory activity in the LPS-induced ALI model by regulating potential biomarkers, including L-phenylalanine, L-tyrosine, and linoleic acid levels in phenylalanine, tyrosine, and tryptophan biosynthesis and linoleic acid metabolism. Thus, the regulation of AJH on phenylalanine, tyrosine, and tryptophan biosynthesis and linoleic acid metabolism may be associated with its alleviatory effects on inflammation responses *in vivo* to improve LPS-induced ALI. Our study initially elucidated serum material basis and effective mechanism of AJH in the ALI treatment by network analysis and untargeted metabolomics. For a better understanding of the molecular mechanism of potential biomarkers and related metabolism pathways, targeted metabolomics, proteomics, and genomics need to be studied further.

## 5 Conclusion

In our study, we integrated metabolomics and network analysis of serum pharmacochemistry and systematically unrevealed the material basis and molecular mechanism of AJH in the ALI treatment. A total of 71 serum components and 18 related metabolites were identified by UPLC-Orbitrap Fusion MS. After network analysis of the aforementioned serum components, five core flavonoid components were selected. Preliminarily, AJH was hypothesized to treat ALI by modulating the core targets, including TNF, TP53, ALB, IL-6, AKT1, MAPK, and TLR4, and related signaling pathways (PI3K-Akt, AGE-RAGE, and JAK-STAT), which laid the foundation for the specific molecular mechanism of ALI treatment by AJH. Meanwhile, metabolomics results showed that AJH was effective in treating ALI by alleviating infiltration of inflammatory cells in alveolar spaces and regulating the expression of inflammatory cytokines. AJH might link to reverse the abnormality of phenylalanine, tyrosine, and tryptophan biosynthesis and linoleic acid metabolism pathways to regulate the concentrations of potential biomarkers to normal levels. Therefore, AJH could alleviate inflammation responses in the ALI treatment.

## Figures and Tables

**Table:** 

**ALI**	Acute lung injury
**AJH**	Ardisiae Japonicae Herba
**LPS**	Lipopolysaccharide
**Dex**	Dexamethasone
**BALF**	Bronchoalveolar lavage fluid
**AGE-RAGE**	Advanced glycation end product–receptor for advanced glycation end product
**PI3K-AKT**	Phosphatidylinositol-3-kinase/protein kinase B
**JAK-STAT**	Tyrosine kinase/signal transducer and transcription activator
**IL**	Interleukin
**CM**	Chinese medicine
**ELISA**	Enzyme-linked immunosorbent assay
**ARRIVE**	Animal Research: Reporting of *in vivo* Experiments
**H&E**	Hematoxylin and eosin
**W/D**	Wet/dry
**QC**	Quality control
**OMIM**	Online Mendelian Inheritance in Man
**BP**	Biological processes
**CC**	Cellular components
**MF**	Molecular functions
**TIC**	Total ion chromatogram
**PCA**	Principal component analysis
**PLS-DA**	Partial least squares-discriminant analysis
**VIP**	Variable importance
**TNF**	Tumor necrosis factor
**TP**	Tumor protein
**ALB**	Albumin
**AKT**	AKT serine/threonine kinase
**MAPK**	Mitogen-activated protein kinase
**TLR**	Toll-like receptor
**SIRS**	Systemic inflammatory response syndrome
**ARDS**	Acute respiratory distress syndrome
**MOF**	Multiple organ failure

## Data Availability

The original contributions presented in the study are included in the article/Supplementary Material; further inquiries can be directed to the corresponding authors.
