# Supplementary material for: Metabolomic profiling combined with network analysis of serum pharmacochemistry to reveal the therapeutic mechanism of Ardisiae Japonicae Herba against acute lung injury
Source: Front Pharmacol. 2023 Jul 24;14:1131479. doi: 10.3389/fphar.2023.1131479 (PMC10405081; doi:10.3389/fphar.2023.1131479)
Supplement: Supplementary file 1 [file DataSheet1.docx]

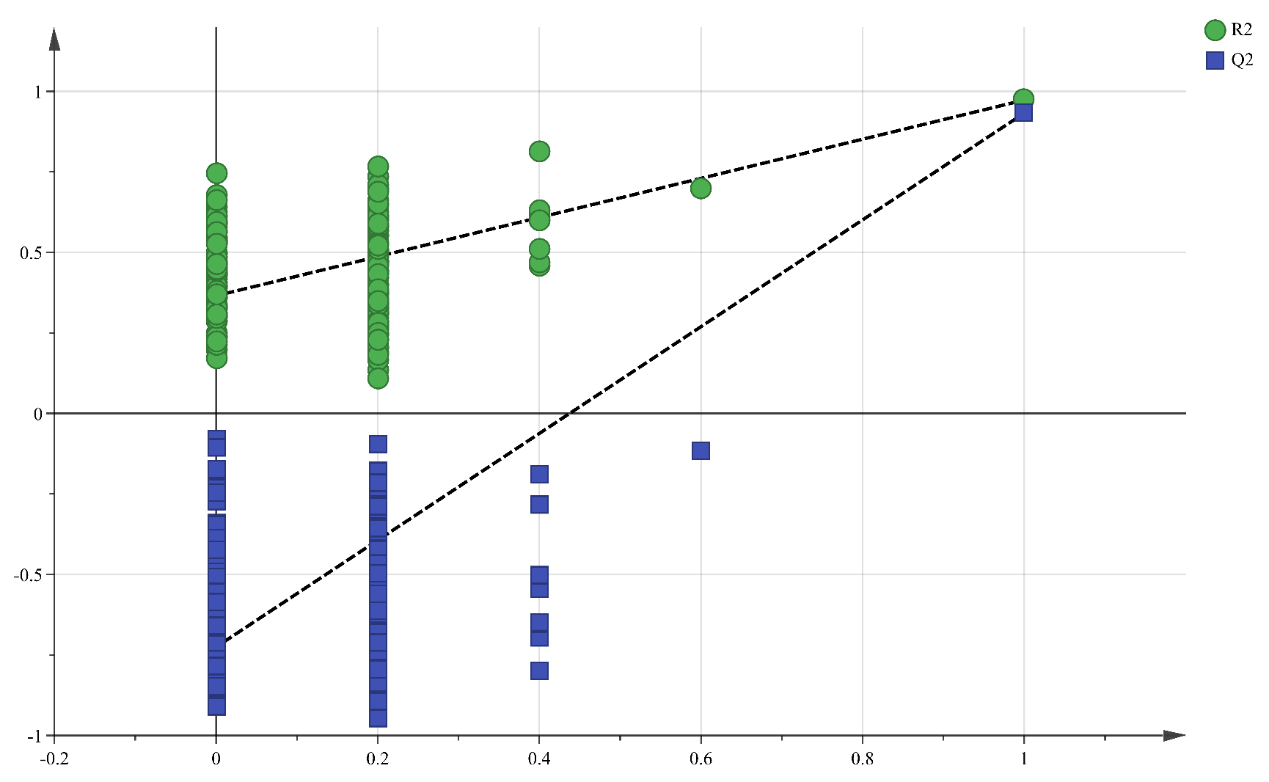


Figure S1. The permutation test of PLS-DA model

Table S1. AJH regulated the expression levels of IL-6 and IL-10 in serum and BALF.

($\bar{X}$*±*SD*,* n=6)

| Group | Serum | | BALF | |
| --- | --- | --- | --- | --- |
|  | IL-6(ng/mL) | IL-10(μg/mL) | IL-6(ng/mL) | IL-10(μg/mL) |
| Control group | 103.00±5.98 | 80.36±2.29 | 96.02±6.56 | 79.44±2.53 |
| LPS group | 142.50±16.03^##^ | 57.35±2.36^##^ | 169.15±9.32^##^ | 58.21±2.25^##^ |
| LPS+AJH-L group | 133.92±7.18 | 62.15±3.48^*^ | 142.25±6.05^**^ | 62.26±2.02^*^ |
| LPS+AJH-M group | 124.67±4.67 | 68.79±3.11^**^ | 127.30±15.50^*^ | 69.75±2.52^**^ |
| LPS+AJH-H group | 114.48±4.48^*^ | 77.62±3.16^**^ | 113.35±5.33^**^ | 74.13±1.13^**^ |
| LPS+Dex group | 110.48±5.99^*^ | 76.92±1.15^**^ | 106.70±8.98^**^ | 72.38±1.16^**^ |
| F Value | 12.131 | 49.820 | 36.130 | 55.865 |

^#^P < 0.05, ^##^P < 0.01 vs. the control group; *P < 0.05, **P < 0.01 vs. the LPS group.

Table S2. Measuring the relevant indexes of lung function analysis in rats. ($\bar{X}$*±*SD*,* n=6)

| Group | Penh | PAU | EF_50_ | PEFb |
| --- | --- | --- | --- | --- |
| Control group | 0.62±0.09 | 0.76±0.10 | 0.85±0.19 | 16.31±3.43 |
| LPS group | 1.29±0.71 | 1.18±0.43 | 1.23±0.28 | 12.76±1.87 |
| LPS+AJH-L group | 0.92±0.51 | 1.01±0.25 | 1.12±0.23 | 14.00±1.30 |
| LPS+AJH-M group | 0.79±0.24 | 0.94±0.34 | 1.08±0.17 | 14.65±2.88 |
| LPS+AJH-H group | 0.71±0.11 | 0.89±0.17 | 1.03±0.35 | 15.74±1.76^*^ |
| LPS+Dex group | 0.76±0.31 | 0.87±0.28 | 0.97±0.15 | 15.41±3.29 |
| F Value | 1.615 | 1.107 | 1.233 | 1.113 |

^#^P < 0.05, ^##^P < 0.01 vs. the control group; *P < 0.05, **P < 0.01 vs. the LPS group.

Table S3. The results of Lung W/D ratio, thymus and spleen indexes. ($\bar{X}$*±*SD*,* n=6)

| Group | Lung W/D ratio | Thymus index | Spleen index |
| --- | --- | --- | --- |
| Control group | 4.26±0.21 | 0.00159±0.00040 | 0.00241±0.00046 |
| LPS group | 4.60±0.11^#^ | 0.00097±0.00012^#^ | 0.00161±0.00014^##^ |
| LPS+AJH-L group | 4.54±0.04 | 0.00115±0.00018 | 0.00189±0.00028 |
| LPS+AJH-M group | 4.47±0.20 | 0.00139±0.00022^**^ | 0.00198±0.00038 |
| LPS+AJH-H group | 4.40±0.20 | 0.00142±0.00012^**^ | 0.00207±0.00012^**^ |
| LPS+Dex group | 4.42±0.44 | 0.00154±0.00013^**^ | 0.00212±0.00032^*^ |
| F Value | 1.124 | 4.491 | 3.188 |

^#^P < 0.05, ^##^P < 0.01 vs. the control group; *P < 0.05, **P < 0.01 vs. the LPS group.

Table S4. The information of GO enrichment analysis.

| **NO.** | **GO pathways** | **Gene Ratio** | **P value** | **Count** |
| --- | --- | --- | --- | --- |
| GOBP-1 | GO:0048534-hematopoietic or lymphoid organ development | 34 | 34.46958406 | 34 |
| GOBP-2 | GO:0002520-immune system development | 34 | 33.51488005 | 34 |
| GOBP-3 | GO:0030097-hemopoiesis | 31 | 30.99469079 | 31 |
| GOBP-4 | GO:0048608-reproductive structure development | 28 | 30.93800825 | 28 |
| GOBP-5 | GO:0061458-reproductive system development | 28 | 30.85145094 | 28 |
| GOBP-6 | GO:1901699-cellular response to nitrogen compound | 31 | 30.26562319 | 31 |
| GOBP-7 | GO:0071417-cellular response to organonitrogen compound | 29 | 28.57559119 | 29 |
| GOBP-8 | GO:0010035-response to inorganic substance | 28 | 28.40130576 | 28 |
| GOBP-9 | GO:0007167-enzyme-linked receptor protein signaling pathway | 29 | 28.02897495 | 29 |
| GOBP-10 | GO:0062197-cellular response to chemical stress | 23 | 27.86620574 | 23 |
| GOBP-11 | GO:0006979-response to oxidative stress | 25 | 27.84740163 | 25 |
| GOBP-12 | GO:0030335-positive regulation of cell migration | 28 | 27.52997877 | 28 |
| GOBP-13 | GO:2000147-positive regulation of cell motility | 28 | 27.0252225 | 28 |
| GOBP-14 | GO:0031347-regulation of defense response | 29 | 26.93172993 | 29 |
| GOBP-15 | GO:0051272-positive regulation of cellular component movement | 28 | 26.76093111 | 28 |
| GOBP-16 | GO:0040017-positive regulation of locomotion | 28 | 26.72081414 | 28 |
| GOBP-17 | GO:0034599-cellular response to oxidative stress | 21 | 26.386389 | 21 |
| GOBP-18 | GO:0032103-positive regulation of response to external stimulus | 25 | 25.45145654 | 25 |
| GOBP-19 | GO:0009725-response to hormone | 29 | 25.34375168 | 29 |
| GOBP-20 | GO:0048732-gland development | 24 | 25.25508873 | 24 |
| GOMF-1 | GO:0004712-protein serine/threonine/tyrosine kinase activity | 20 | 18.48489349 | 20 |
| GOMF-2 | GO:0004672-protein kinase activity | 21 | 17.76443261 | 21 |
| GOMF-3 | GO:0004713-protein tyrosine kinase activity | 13 | 16.2971942 | 13 |
| GOMF-4 | GO:0016773-phosphotransferase activity, alcohol group as acceptor | 21 | 16.22032454 | 21 |
| GOMF-5 | GO:0016301-kinase activity | 21 | 15.52889697 | 21 |
| GOMF-6 | GO:0019900-kinase binding | 21 | 15.05828472 | 21 |
| GOMF-7 | GO:0019902-phosphatase binding | 13 | 14.2953015 | 13 |
| GOMF-8 | GO:0019901-protein kinase binding | 19 | 13.69303611 | 19 |
| GOMF-9 | GO:0008134-transcription factor binding | 18 | 13.64902065 | 18 |
| GOMF-10 | GO:0061629-RNA polymerase II-specific DNA-binding transcription factor binding | 15 | 13.59443879 | 15 |
| GOMF-11 | GO:0140297-DNA-binding transcription factor binding | 16 | 12.91245917 | 16 |
| GOMF-12 | GO:0001221-transcription coregulator binding | 10 | 12.42790949 | 10 |
| GOMF-13 | GO:0019199-transmembrane receptor protein kinase activity | 9 | 12.09494213 | 9 |
| GOMF-14 | GO:0004714-transmembrane receptor protein tyrosine kinase activity | 8 | 11.36711781 | 8 |
| GOMF-15 | GO:0016922-nuclear receptor binding | 10 | 11.35912407 | 10 |
| GOMF-16 | GO:0019903-protein phosphatase binding | 10 | 11.08608702 | 10 |
| GOMF-17 | GO:0001223-transcription coactivator binding | 7 | 10.87612166 | 7 |
| GOMF-18 | GO:0042803-protein homodimerization activity | 16 | 10.44216381 | 16 |
| GOMF-19 | GO:0019904-protein domain specific binding | 16 | 10.43282491 | 16 |
| GOMF-20 | GO:0005126-cytokine receptor binding | 11 | 9.745706226 | 11 |
| GOCC-1 | GO:0045121-membrane raft | 17 | 16.77863329 | 17 |
| GOCC-2 | GO:0098857-membrane microdomain | 17 | 16.75632464 | 17 |
| GOCC-3 | GO:0098552-side of membrane | 20 | 14.91867814 | 20 |
| GOCC-4 | GO:0009897-external side of plasma membrane | 16 | 13.05545819 | 16 |
| GOCC-5 | GO:0005667-transcription regulator complex | 15 | 11.39141462 | 15 |
| GOCC-6 | GO:0043235-receptor complex | 15 | 10.81222449 | 15 |
| GOCC-7 | GO:0031983-vesicle lumen | 12 | 10.11311457 | 12 |
| GOCC-8 | GO:0060205-cytoplasmic vesicle lumen | 11 | 8.929768091 | 11 |
| GOCC-9 | GO:0005925-focal adhesion | 12 | 8.863087608 | 12 |
| GOCC-10 | GO:0030055-cell-substrate junction | 12 | 8.759715323 | 12 |
| GOCC-11 | GO:0034774-secretory granule lumen | 10 | 7.798646335 | 10 |
| GOCC-12 | GO:0044853-plasma membrane raft | 7 | 7.627876817 | 7 |
| GOCC-13 | GO:0005901-caveola | 6 | 7.03709427 | 6 |
| GOCC-14 | GO:0090575-RNA polymerase II transcription regulator complex | 8 | 6.531014483 | 8 |
| GOCC-15 | GO:0031012-extracellular matrix | 11 | 6.434056334 | 11 |
| GOCC-16 | GO:0030312-external encapsulating structure | 11 | 6.426554646 | 11 |
| GOCC-17 | GO:0005788-endoplasmic reticulum lumen | 8 | 5.712595042 | 8 |
| GOCC-18 | GO:0031091-platelet alpha granule | 5 | 5.322783133 | 5 |
| GOCC-19 | GO:0009898-cytoplasmic side of plasma membrane | 6 | 5.132545661 | 6 |
| GOCC-20 | GO:0098562-cytoplasmic side of membrane | 6 | 4.725809096 | 6 |

Table S5. The information of KEGG enrichment analysis.

| **NO.** | **KEGG Signaling Pathways** | **Gene Ratio** | **P value** | **Count** |
| --- | --- | --- | --- | --- |
| 1 | hsa05200: Pathways in cancer | 45 | 56.92256285 | 45 |
| 2 | hsa05161: Hepatitis B | 24 | 35.01191429 | 24 |
| 3 | hsa05167: Kaposi sarcoma-associated herpesvirus infection | 24 | 33.02428095 | 24 |
| 4 | hsa05417: Lipid and atherosclerosis | 22 | 28.31245329 | 22 |
| 5 | hsa04151: PI3K-Akt signaling pathway | 25 | 28.18264775 | 25 |
| 6 | hsa05166: Human T-cell leukemia virus 1 infection | 22 | 27.99630056 | 22 |
| 7 | hsa04933: AGE-RAGE signaling pathway in diabetic complications | 18 | 27.75536541 | 18 |
| 8 | hsa05205: Proteoglycans in cancer | 21 | 27.00769279 | 21 |
| 9 | hsa05145: Toxoplasmosis | 18 | 26.79959968 | 18 |
| 10 | hsa05152: Tuberculosis | 19 | 24.65110449 | 19 |
| 11 | hsa01521: EGFR tyrosine kinase inhibitor resistance | 15 | 23.49618195 | 15 |
| 12 | hsa04659: Th17 cell differentiation | 16 | 23.2054858 | 16 |
| 13 | hsa05171: Coronavirus disease - COVID-19 | 19 | 22.5028301 | 19 |
| 14 | hsa05215: Prostate cancer | 15 | 22.05435575 | 15 |
| 15 | hsa04630: JAK-STAT signaling pathway | 17 | 21.99130513 | 17 |
| 16 | hsa01522: Endocrine resistance | 15 | 21.98310067 | 15 |
| 17 | hsa05142: Chagas disease | 15 | 21.70583166 | 15 |
| 18 | hsa04625:C-type lectin receptor signaling pathway | 15 | 21.57161981 | 15 |
| 19 | hsa05162: Measles | 16 | 21.36166862 | 16 |
| 20 | hsa05235: PD-L1 expression and PD-1 checkpoint pathway in cancer | 14 | 20.70205388 | 14 |
